# TLR 2 and 4 Responsiveness from Isolated Peripheral Blood Mononuclear Cells from Rats and Humans as Potential Chronic Pain Biomarkers

**DOI:** 10.1371/journal.pone.0077799

**Published:** 2013-10-30

**Authors:** Yuen H. Kwok, Jonathan Tuke, Lauren L. Nicotra, Peter M. Grace, Paul E. Rolan, Mark R. Hutchinson

**Affiliations:** 1 Discipline of Pharmacology, School of Medical Sciences, University of Adelaide, Adelaide, South Australia, Australia; 2 School of Mathematical Sciences, University of Adelaide, Adelaide, South Australia, Australia; 3 Department of Psychology and The Center for Neuroscience, University of Colorado at Boulder, Boulder, Colorado, United States of America; 4 Pain and Anaesthesia Research Clinic, University of Adelaide, Adelaide, South Australia, Australia; 5 Pain Management Unit, Royal Adelaide Hospital, Adelaide, South Australia, Australia; 6 Discipline of Physiology, School of Medical Sciences, University of Adelaide, Adelaide, South Australia, Australia; University of Arizona, United States of America

## Abstract

**Background:**

Chronic pain patients have increased peripheral blood mononuclear cell Interkeukin-1β production following TLR2 and TLR4 simulation. Here we have used a human-to-rat and rat-to-human approach to further investigate whether peripheral blood immune responses to TLR agonists might be suitable for development as possible systems biomarkers of chronic pain in humans.

**Methods and Results:**

Study 1: using a graded model of chronic constriction injury in rats, behavioral allodynia was assessed followed by *in vitro* quantification of TLR2 and TLR4 agonist-induced stimulation of IL-1β release by PBMCs and spinal cord tissues (n = 42; 6 rats per group). Statistical models were subsequently developed using the IL-1β responses, which distinguished the pain/no pain states and predicted the degree of allodynia. Study 2: the rat-derived statistical models were tested to assess their predictive utility in determining the pain status of a published human cohort that consists of a heterogeneous clinical pain population (n = 19) and a pain-free population (n = 11). The predictive ability of one of the rat models was able to distinguish pain patients from controls with a ROC AUC of 0.94. The rat model was used to predict the presence of pain in a new chronic pain cohort and was able to accurately predict the presence of pain in 28 out of the 34 chronic pain participants.

**Conclusions:**

These clinical findings confirm our previous discoveries of the involvement of the peripheral immune system in chronic pain. Given that these findings are reflected in the prospective graded rat data, it suggests that the TLR response from peripheral blood and spinal cord were related to pain and these clinical findings do indeed act as system biomarkers for the chronic pain state. Hence, they provide additional impetus to the neuroimmune interaction to be a drug target for chronic pain.

## Introduction

Pain as defined by the International Association for the Study of Pain is an unpleasant sensory and emotional experience associated with actual or potential tissue damage, or described in terms of such damage. Pain is a subjective experience, and hence it is conventionally assessed by patient reports, sometimes with added rating scales. Preclinical animal pain research cannot use such measures, but instead relies on behavioral responses to infer the pain experience. There is a large failure rate in clinical translation of therapies that are efficacious in standard preclinical animal studies, possibly in part because of these different assessments [1]. Biomarkers that reflect pain biology and which could be used in both preclinical animal and clinical human studies have the potential for improving translational success in pain research. Additionally, practical human pain biomarkers have potential uses in enriching clinical trial populations, assisting in the selection of patient treatment and monitoring treatment efficacy [2].

Development of pain biomarkers is problematic because of difficulty in accessing the central nervous system (CNS) where the chronic pain pathology likely resides. Although neuroimaging has emerged as a potential biomarker for chronic pain, by providing “pain signatures” of the brain [3]–[6], there are several limitations to its usage [7]. Instead, we have sought evidence that peripheral tissues reflect functional changes of the CNS and hence have the potential to be accessible human pain biomarkers.

Over the past 20 years substantial evidence has accumulated indicating the involvement of non-neuronal cells playing a pivotal role in chronic pain. In particular, the immunocompetent cells of the CNS, glia, respond to pain signals releasing additional pro-nociceptive proinflammatory mediators that in turn sensitize neighboring neurons and glia facilitating the heightened pain state [8]–[12]. Interestingly, this research points to attenuating proinflammatory glial activation is a promising new target for the treatment of neuropathic pain, as drugs that attenuate pro-inflammatory glial activation results in a reduction in allodynia [13]–[16].

A key mediator in the initiation of proinflammatory glial reactivity associated with chronic pain is Toll Like Receptors (TLRs). TLRs are an innate immune receptor family that recognize danger-associated molecular patterns (DAMP) and pathogen-associated molecular patterns [17]. Activation of TLRs causes the production of pro-inflammatory mediators such as pro-inflammatory cytokines (such as IL-1β) [9]. It is clear from preclinical models that glia assume a proinflammatory reactive state following activation by TLRs and that blockade of glial TLRs significantly reduces experimentally induced neuropathic pain [18]–[20]. Interestingly, we have recently demonstrated that in chronic pain patients, peripheral blood mononuclear cells (PBMCs) also have increased TLR2 and TLR4 responsiveness compared with pain-free participants [21], suggesting that this could be a potential pain biomarker.

However, given the cross-sectional nature of the human data which hinders cause-and-effect analysis, we have sought whether similar findings occur in a prospective graded animal model to support that interpretation that this biomarkers lie on the causal pathway rather than being bystander effects. Additionally we have sought whether a biomarker panel based on TLR-induced IL-1β production by PBMCs can distinguish pain from non-pain states in two separate clinical pain populations (medication overuse headache and sciatica) to explore potential clinical utility.

## Materials and Methods

### Study 1: Graded Chronic Constriction Injury Surgery and Sample Preparation

#### Animals

Pathogen-free adult male Sprague–Dawley rats (300–350 g; University of Adelaide, Laboratory Animal Services, Waite Campus, Urrbrae, Australia) were used in all experiments. Rats were housed in a temperature-controlled (18–21°C) and light-controlled (12 h light/dark cycle; lights on at 07∶00 h) rooms with standard rodent chow and water available *ad libitum.* Animals were habituated to the holding facility for 1 week prior to experimentation. All procedures were approved by the Animal Ethics Committee of the University of Adelaide and were conducted in accordance with the NHMRC Australian Code of Practice for the Care and Use of Animals for Scientific Purposes.

#### Surgery

A graded neuropathic pain model, the “Grace model” was used [22]. Surgery was conducted under isofluorane (3% oxygen) anaesthesia. Briefly, the sciatic nerve was exposed at the mid-thigh level of the left leg as previously described [23]. Between zero and 4 sterile chromic gut sutures (cuticular 4–0 chromic gut, FS-2; Ethicon, Somerville, NJ, USA) were loosely tied around the gently isolated sciatic nerve to produce varying degrees of allodynia. Once the superficial muscle overlying the nerve was sutured, the animals had varying numbers of chromic gut suture (equivalent length) placed in the subcutaneous space. For sham treatment, the sciatic nerve was identically exposed and isolated but not tied. Animals were monitored postoperative (PO) until fully ambulatory prior to the return of their cage and checked daily for signs of infection. No such cases occurred in this study.

#### Experimental groups and design

Experimental groups used in the Grace model were also selected in this study. The sciatic nerve was loosely ligated with chromic gut sutures, with the number of perineural sutures indicated by the designation N0, N1, N2 or N4. Additional pieces of chromic gut designated S4, S3, S2 or S0 respectively were also placed in the subcutaneous space, to keep the total number of ligatures to 4, in order to keep the non-specific immunological stimulus constant between the groups. This model has been shown to produce graded neuropathic pain in relation to the number of ligatures around the nerve. Two additional groups (N1S0 and N2S0) with only ligatures to the sciatic nerve were also introduced to examine only neuronal insults. For the sham control the nerve was isolated but there was no exposure to chromic gut. N0S4 was a control group for the presence of chromic gut. The experimental groups (6 rats/group) were N0S0 (sham control), N0S4, N1S0, N1S3, N2S0, N2S2 and N4S0.

#### Behavioral testing: von frey test

Rats were habituated for at least three sessions (60 min) to the test environment prior to von Frey testing. Testing was performed blinded with respect to the experimental group. The von Frey test was performed within the sciatic innervation area of the hind paw. Assessments were at baseline, PO day 3, 7, 10 and day of cull and the development of allodynia was assessed. Animals were followed to at least PO day 18 to ensure the neuropathic pain was well established. A logarithmic series of ten calibrated Semmes-Weinstein monofilaments (von Frey hairs; Stoelting, Wood Dale, IL, USA) were applied randomly to the left hind paw to determine the stimulus intensity threshold stiffness required to elicit a paw withdrawal response. Log_10_ (milligrams×10) hair stiffness ranged from 3.61 (0.407 g) to 5.18 (15.136 g). The behavioral responses were used to calculate the 50% paw withdrawal threshold (absolute threshold), by fitting a Gaussian integral psychometric function using a maximum-likelihood fitting method using the program PsychoFit [24]. This fitting method allows parametric analyses that otherwise would not be appropriate.

#### Peripheral blood and spinal cord collection

On the day of cull (at least PO 18 day), rats were anesthetized with sodium pentobarbital and blood (approximately 7 mL) was collected via cardiac puncture into tubes containing EDTA. The rat was then transcardially perfused with 15 ml of chilled 0.9% isotonic saline and the lumbar spinal cord was quickly removed and dissected into 3 equal lengths. The isolated spinal cord was incubated for 20 h at 37°C, 5% CO_2_ in a humidified environment (Thermoline Scientific, Australia). Added to the incubation medium were either: 10 µg/mL of TLR2 agonist synthetic triacylated lipoprotein (Pam3CSK4) or 10 µg/mL of TLR4 agonist lipopolysaccharide (LPS) from Sigma-Aldrich (Castle Hill, NSW, Australia) or RPMI medium only (control).

#### Stimulation of rat peripheral blood mononuclear cells (PBMCs) and plasma collection

PBMCs were isolated using Optiprep Sigma-Aldrich (Castle Hill, NSW, Australia) as directed by the manufacturer using the mixer flotation method. Plasma was also collected and stored at −70°C until the ELISA. Isolated cells were diluted to 1×10^6^ cells·ml^−1^ in enriched RPMI 1640 (10% fetal calf serum and 1% penicillin) and plated into 96 well plates (Nunc, Roskilde, Denmark) (100 µl per well). When insufficient cells were obtained (5 rats), data were adjusted to 1×10^6^ cells (by multiplication of the factor to obtain a response for IL-1β 1×10^6^ cells). A range of concentrations was added into the wells, TLR2 agonist (Pam_3_CSK_4_) from 10 ng·ml^−1^ to 1 µg·ml^−1^ and TLR4 agonist (LPS) from 10 ng·ml^−1^ to 10 µg·ml^−1^. Control wells minus the TLR agonist were also included. Plates were incubated for 20 h at 37°C, 5% CO_2_ in a humidified environment (Thermoline Scientific, Australia).

#### Spinal cord sample preparation

Briefly, after 20 h of incubation the supernatant of the spinal cord was stored at −80°C until assay. The spinal cord sections were removed and sonicated using a Labsonic 1510 probe sonicator (B. BRAUN, Melsungen, Germany) in ice-cold extraction buffer containing Iscove’s medium with 5% FCS and a cocktail enzyme inhibitor (including: 100 mM amino-n-caproic acid, 10 mM EDTA, 5 mM benzamidine-HCL, and 0.2 mM phenylmethylsulfonyl fluoride) all obtained from Sigma-Aldrich (Castle Hill, NSW, Australia). Sonicated samples were centrifuged with the supernatant and stored at −70°C until assay.

### Study 2: Chronic Pain and Pain-free Participants

#### Study participants

The data presented here was obtained from 1 published study [21] and 2 unpublished clinical studies. Ethical approval was obtained from the Human Research Ethics Committee of the Royal Adelaide Hospital, Adelaide, South Australia. All studies were conducted at the Pain and Anaesthesia Research Clinic (PARC), Royal Adelaide Hospital, Adelaide, Australia.

All participants gave written informed consent to participate after a detailed oral explanation of the study. All participants were paid for their inconvenience upon completion of the study. Chronic pain patients were recruited from the PARC volunteer database, by public advertisements and from a pain management unit. Healthy participants were recruited from the PARC’s healthy participant database. Sixty-four participants were recruited and participants were divided into 2 cohorts: published cohort (consisted of participants from a previous study [21]) and an expanded cohort.

The published cohort consisted of chronic pain participants and pain-free participants. Chronic pain participants had to experience pain at least five days a week and for at least 3 months. The pain-free participants had no clinically significant chronic pain and were not taking opioids or other analgesics. The expanded cohort consisted of mainly unilateral sciatica and medication-overuse headache participants. Unilateral sciatica participants had to experience pain at least five days a week and for at least 3 months. For the medication-overuse headache participants, the inclusion criteria included regular use for at least 3 months of opioid-containing analgesics (10≥ days per month) headache present on at least 15 days/month (for at least 2 months), headache developed or markedly worsened during medication-overuse and primary indication for analgesics is a headache disorder.

There was no minimum pain score for eligibility. Chronic pain patients from both cohorts could be taking ongoing opioid therapy or not on any chronic opioid therapy. For all participants the key inclusion criteria were the following: aged between 18 and 65 years, be in good general health (other than chronic pain patients) without clinically significant renal, hepatic, cardiac or other diseases. Key exclusion criteria were: use of any immunosuppressant drugs (e.g. azathioprine); presence of an active inflammatory process; a clinically significant infection in the previous 4 weeks; a positive urine screen for illicit drugs (except for prescribed opioids), pregnancy and/or lactation, and have a known history of hepatitis B, C or HIV.

#### Human blood collection and PBMCs isolation

On the study day, information on pain history and medication use was recorded. Twenty-seven ml of blood were collected into tubes containing EDTA and the same procedure mentioned previously in “Stimulation of rat PBMCs and plasma collection” was performed. Sufficient cells were obtained from all participants and plasma was not collected in humans. A range of concentrations of TLR agonists were added into the wells in triplicate, Pam3CSK4 from 13 pg·ml^−1^ to 1 µg·ml^−1^ (Sigma) and LPS from 6 pg·ml^−1^ to 10 µg·ml^−1^ (Sigma). Control wells minus the TLR agonist were also included.

### Rat and Human IL-1β Assay

IL-1β level was determined by a commercially available ELISA (rat IL-1β ELISA; eBioscience, San Diego, CA and for human IL-1β ELISA; BD Bioscience, Australia). For the rat’s ELISA kit, the manufacturer’s instructions were modified by extending the standard curve from 39 pg/mL to 5 pg/mL so that lower concentration of IL-1β could be detected. The extended standard curve was accepted for each ELISA when the R-square (goodness of fit) was above 0.99. For the human’s ELISA kit the IL-1β levels were determined according to the manufacturer’s instructions. UV absorbance was quantified on a BMG PolarStar microplate reader (BMG Labtechnologies, Offenburg, Germany) at 450 nm with absorbance at 570 nm subtracted. The modified limit of quantification of 5 pg·ml^−1^ was used for the rat’s ELISA kit and for the human’s ELISA kit the manufacturer’s limit of quantification of 0.8 pg·ml^−1^ was used.

### Study 1 Development of Models from Peripheral and Central Obtained IL-1β Released from Post Graded CCI Rats

#### Overview of modeling

**Figure 1:**
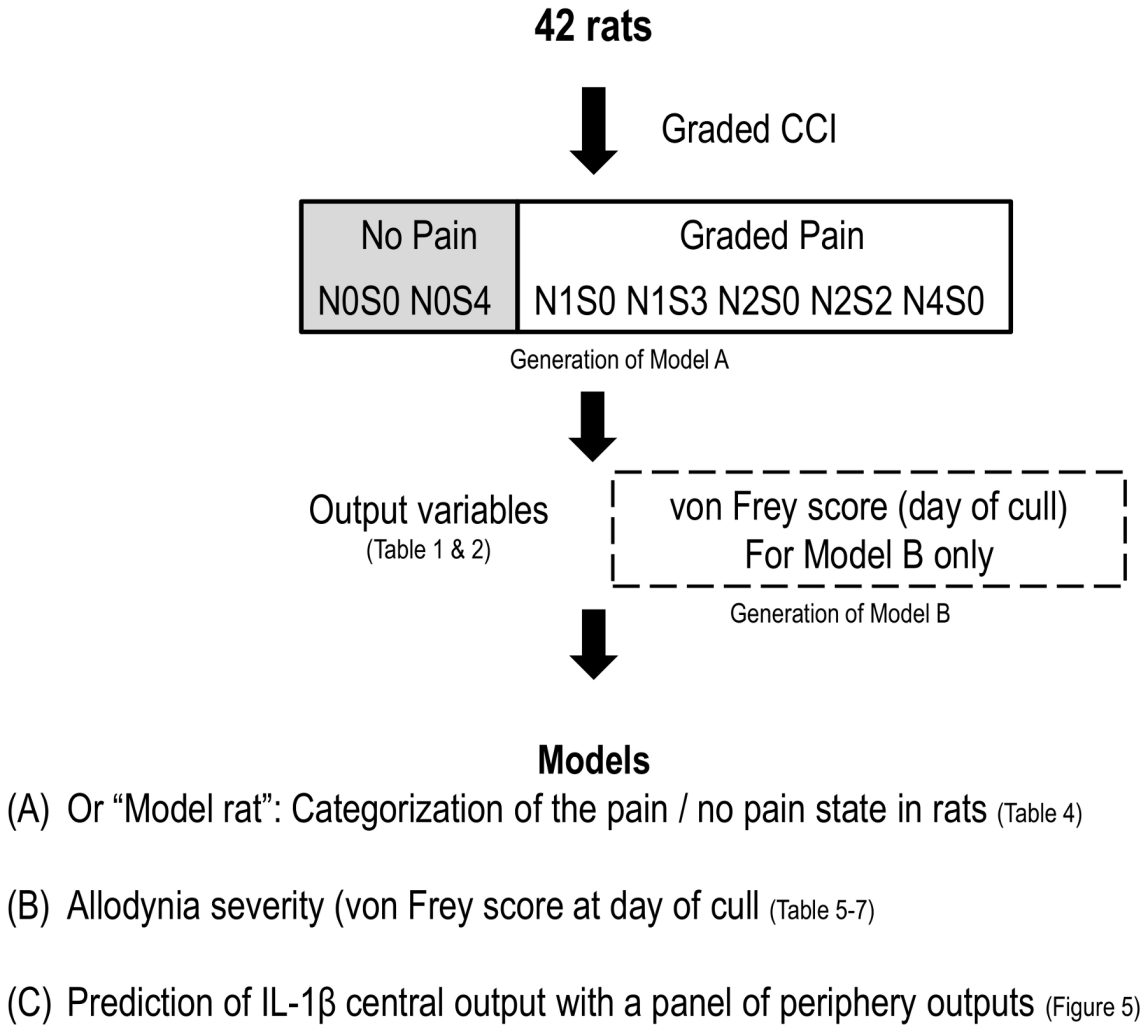
Overview of the generation of models in post graded CCI rats.

The overview of the modeling is summarize in Figure 1. All the collected outputs from the rat (presented in Table 1 and 2.) were imported into the statistical computing environment R (R Development Core Team, 2007). In order to determine whether models constructed with the collected output variables allow: (A) categorization of the pain/no pain states in rats (B) the detection of the allodynia severity in rats (C) whether in rats, central outputs can be predicted with peripheral outputs. The generalized linear model (glm) and the R function stepAIC were used to generate models. StepAIC function [25] performs stepwise model selection (backward and forward selection) using the Akaike information criterion (AIC) as a variable selection criterion. The functions glm and stepAIC are both found in the Modern Applied Statistics with S (MASS) package (From the statistical software R; www.r-project.org).

**Table 1:** Summary of variables collected from rats post CCI in the periphery region.

Variables									P- value
Peripheral (PBMCs)		N0S0	N0S4	N1S3	N2S2	N4S0	N1S0	N2S0	
**Non-stimulated**	**Plasma IL-1β (pg/mL)**	1.7±0.4	2.7±0.7	1.4±0.4	3.3±0.3	10±5.3	4±0.3	5.5±1.7	0.03
	**Cell count** **(^∧^6 cells/mL)**	3.4±1.8*	7.4±1.5	9.2±2.8*#	3.5±0.8	5.9±1	1.8±0. 7**#**	5.6±2.1	0.004
	**Cells IL-1β (pg/mL)**	6.3±1.7	4.9±0.6	5.7±1	7.5±2.8	4.6±0.4	4.8±1.1	4.7±0.4	1
**Pam3CSK4stimulated (TLR2)** **IL-1β (pg/mL)**	**Minimum**	2.3±0.7	3.1±0.9	2.5±0.4	3.6±0.5	2.2±0.7	3.7±0.7	3.8±0.7	0.5
	**Maximum**	8.4±1.8	13.9±4	14.5±3.2	12.7±3.5	9.5±1.4	8.6±2.4	10.5±2.3	0.7
	**Slope**	−0.6±0.5	−2.1±1.7	1.1±1.7	0.3±0.9	−0.1±0.8	−0.09±0.6	−1.9±1.4	0.02
	**Intercept**	5.4±1.1	8.7±2.6	5.8±0.6	7±1	5.6±0.5	5.7±0.9	6.4±1.04	0.4
**LPS stimulated (TLR4) IL-1β** **(pg/mL)**	**Minimum**	12±3.3	14.5±6.1	26.4±10.2	23.6±7.2	16±3.1	12.8±4.7	16±7.3	0.2
	**Maximum**	24.5±6.8	31.4±10.6	70.5±39.7	65.4±19.1	27.7±5.8	41.5±19	45.3±25.3	0.7
	**Slope**	3.7±1.8	2.8±2	−17.4±17.5	11.8±7.5	3.2±1.9	9.2±4.8	8.9±9	0.5
	**Intercept**	13.7±3	20.1±6.8	60.7±35.8	28.6±7.7	18.2±3.1	16.3±6.1	18.9±5.6	0.8
		**Control**	**Neuronal and Subcutaneous**			**Neuronal**			
		**Experimental Groups**							

Data are presented as mean ± SEM. Peripheral blood mononuclear cells (PBMCs) were isolated from 41 rats and stimulated with LPS (10 ng·ml^−1^ to 10 µg·ml^−1^) and with Pam3CSK4 (from 10 ng·ml^−1^ to 1 µg·ml^−1^) for 20 h. No PBMCs could be obtained from 1 rat in N2S0. Plasma were collected from 42 rats. Cell counts were normalized by log transformation and analyzed using one-way ANOVA followed by Bonferroni. It should be noted the following rats did not have enough PBMCs therefore the rat’s PBMCs reactivity were normalize to 10^∧^6 cells in the following rats: 2 rats from N0S0, 3 rats from N1S0 and 1 rat from N2S0. Higher cell counts were detected in experimental group N1S3 vs. N0S0 (indicated with *, *P* = 0.049) and N1S0 vs. N1S3 (Indicated with #, *P* = 0.035). Data (except cell count) were analyzed using Kruskal-Wallis one-way ANOVA.

**Table 2:** Summary of variables collected from rats post CCI in the central region.

Variables									P- value
Central (lumbar spinal cord)		N0S0	N0S4	N1S3	N2S2	N4S0	N1S0	N2S0	
**Non-stimulated IL-1β (pg/mL)**	**Spinal cord supernatant**	4.4±0.4	34.4±24.9	5.1±0.8	9.3±2.9	10.4±5.1	16.5±7.5	7.3±1.9	0.7
	**Spinal cord**	66.6±11.4	60±10.6	55.4±15.2	50.4±10.4	50.5±10.9	50±15.6	67.4±14.2	0.8
**Pam3CSK4stimulated (TLR2)** **IL-1β (pg/mL)**	**Spinal cord supernatant**	5.2±0.6	7.1±1.5	6.1±1.4	7±2	6±1.3	9.2±2.7	6.7±1.5	0.9
	**Spinal cord**	62±12	42.6±2.	50.5±12.2	64.4±12.8	79±7.2	64.8±10.6	83.5±13.1	0.2
**LPS stimulated (TLR4) IL-1β** **(pg/mL)**	**Spinal cord supernatant**	6.8±0.7	7.7±2.5	11.1±4.6	9±1.8	7±1.3	11.8±5.5	6.2±0.8	0.9
	**Spinal cord**	48.5±7.1	50.3±7.1	61.6±10.3	81.8±16.9	51.7±8.2	67.2±11.8	66.3±8.9	0.6
		**Control**	**Neuronal and Subcutaneous**			**Neuronal**			
		**Experimental Groups**							

Data are presented as mean ± SEM. Spinal cord sections were collected from 42 rats and stimulated with LPS at 100ug/mL and Pam3CSK4 at 100 ug/mL. Experimental groups were analyzed using Kruskal-Wallis one-way ANOVA.

The glm function assesses how much each output variable contributes to a response; the responses in question were (A) pain, (B) von Frey score and (C) spinal cord IL-1β output (Figure 1). The stepAIC function was used to refine the model by identifying specific output variables that contributed the most to the model and removed others that added no value to the model.

#### Grouping of output variables

**Figure 2:**
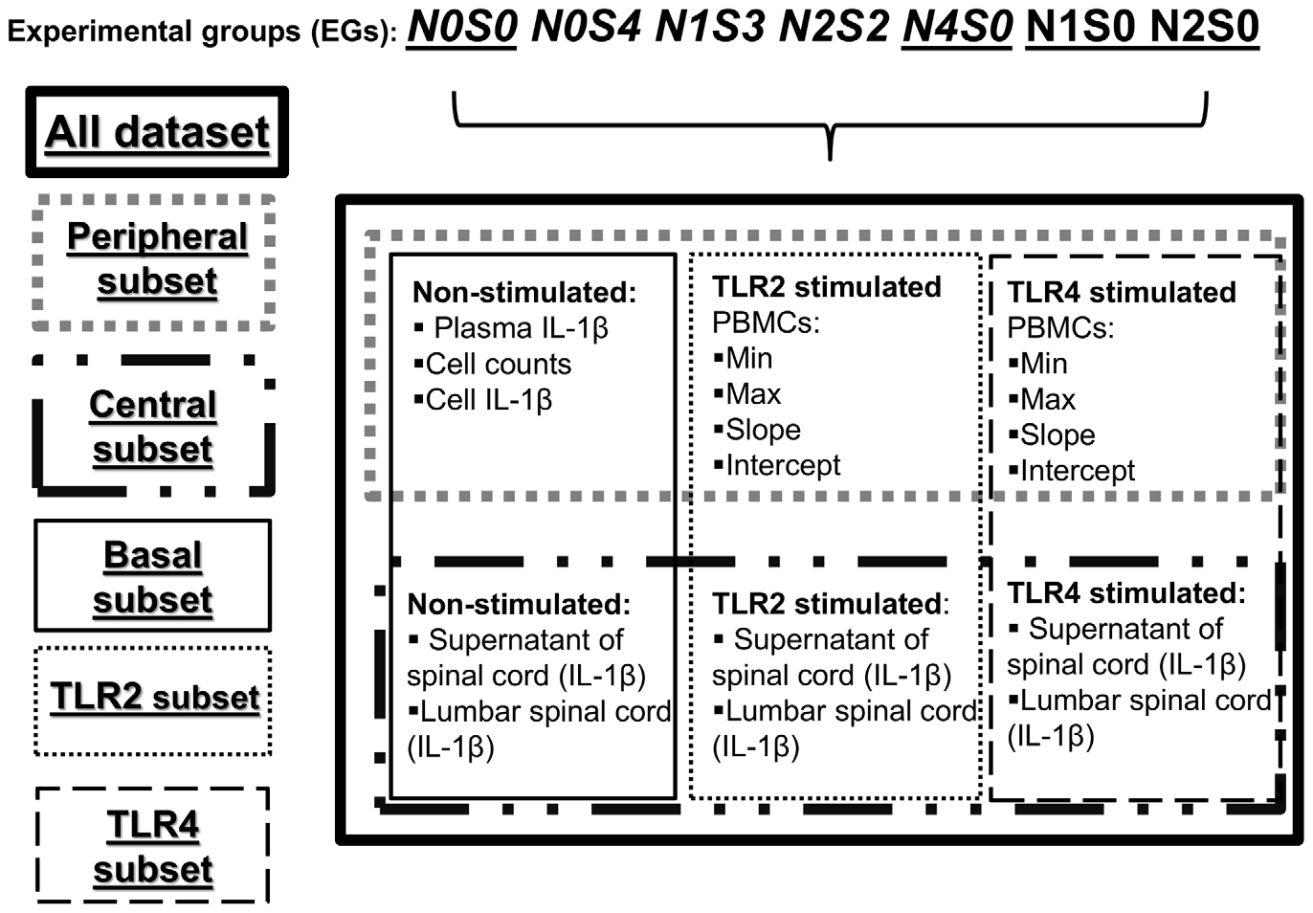
The schematic representation of the breakdown of data according to anatomical location and stimulations. Experimental groups N0S0 and N0S4 are present in both “neuronal and subcutaneous” (italics) and in “neuronal” (underline).

To further dissect which output variables were needed to create the best model to predict the presence and severity of allodynia. The output variables were divided according to anatomical locations and by stimulations as outlined in Figure 2. “Dataset” contained all output variables collected from all anatomical locations and from all stimulations. Whereas subsets contained specific output variables group from either specific location (e.g. Central subset only consist output variables obtained from central region) or from specific stimulation (e.g. TLR2 subset consist output variables stimulated only with TLR2 agonists). The 5 subsets were Peripheral, Central, Basal, TLR2 and TLR4.

To explore the interaction between the effects of nerve alone and combined suture placement, experimental groups were also divided into “**Neuronal and subcutaneous**” (N0S0, N0S4, N1S3, N2S2 and N4S0) and “**Neuronal**” (N0S0, N1S0, N2S0 and N4S0) groups. Within the group, the output variables were further divided into the 5 different subsets as mentioned above.

#### Best model A selection: To predict the presence of pain

N0S0 and N0S4 were experimental groups which considered to have no pain (assigned as 0) because on the day of cull the behavior score indicated there were no group differences between N0S0 (sham) and N0S4. The 5 experimental groups considered to have pain were assigned as 1 and consists of N1S0, N1S3, N2S0, N2S2 and N4S0 (see Figure 1). The glm function was used to predict the presence of pain for the following datasets and subsets from **all** experimental groups:

- **All** dataset and the following 5 subsets: **Peripheral**, **Central**, **Basal, TLR2** and **TLR4**

The stepAIC function was performed to select output variables that contributed significantly to the refined model. Receiver Operating Characteristic (ROC) curves were generated from the refined model and the area under the curve was calculated. One-way ANOVA was used to compare the model generated from the **All** dataset with the 5 subsets (see Figure 1).

The same process was repeated with the **Neuronal and subcutaneous** and **Neuronal** only experimental groups and their corresponding 5 subsets (e.g. Peripheral).

#### Best model B selection: To predict the severity of allodynia

The glm function was used to predict the severity of allodynia (von Frey score at day of cull) and the stepAIC function was used to identify the refined model for **All** experimental groups:

- **All** dataset and the following 5 subsets: **Peripheral**, **Central**, **Basal**, **TLR2** and **TLR4**

A Pearson correlation was chosen to determine the relationship between the actual von Frey score and the data predicted by the refined model. The adjusted R-square was used as it takes into account the number of variables introduced to the refined model. One-way ANOVA was used to compare the model generated from the **All** dataset with the 5 subsets (see Figure 2) to determine which models is a better predictor of the severity of allodynia.

The same process was repeated with the **Neuronal and subcutaneous** and **Neuronal** only experimental groups and their corresponding 5 subsets (e.g. Peripheral).

#### Best model C selection: Prediction of IL-1β central output by models generated from peripheral outputs

The glm function and the stepAIC function were used to generate the refined model to predict the basal spinal cord supernatant IL-1β with output variables obtained from the **Peripheral** subset (from **All** and for **Neuronal and subcutaneous** experimental groups). A Pearson correlation was used to determine the relationship between the predicted values (from the refined model) with the actual IL-1β released from the basal spinal cord supernatant. The adjusted R-square was used and the same procedure was used to generate the model to predict the IL-1β released from the lumbar spinal cord supernatant (post TLR2 and TLR4) and the lumbar spinal cord (basal, post TLR2 and TLR4) response.

### Study 2 Development of Model D to Predict the Presence of Pain in Humans (Chronic Pain and Pain-free Participants) and Compare Models Generated from Study 1

**Figure 3:**
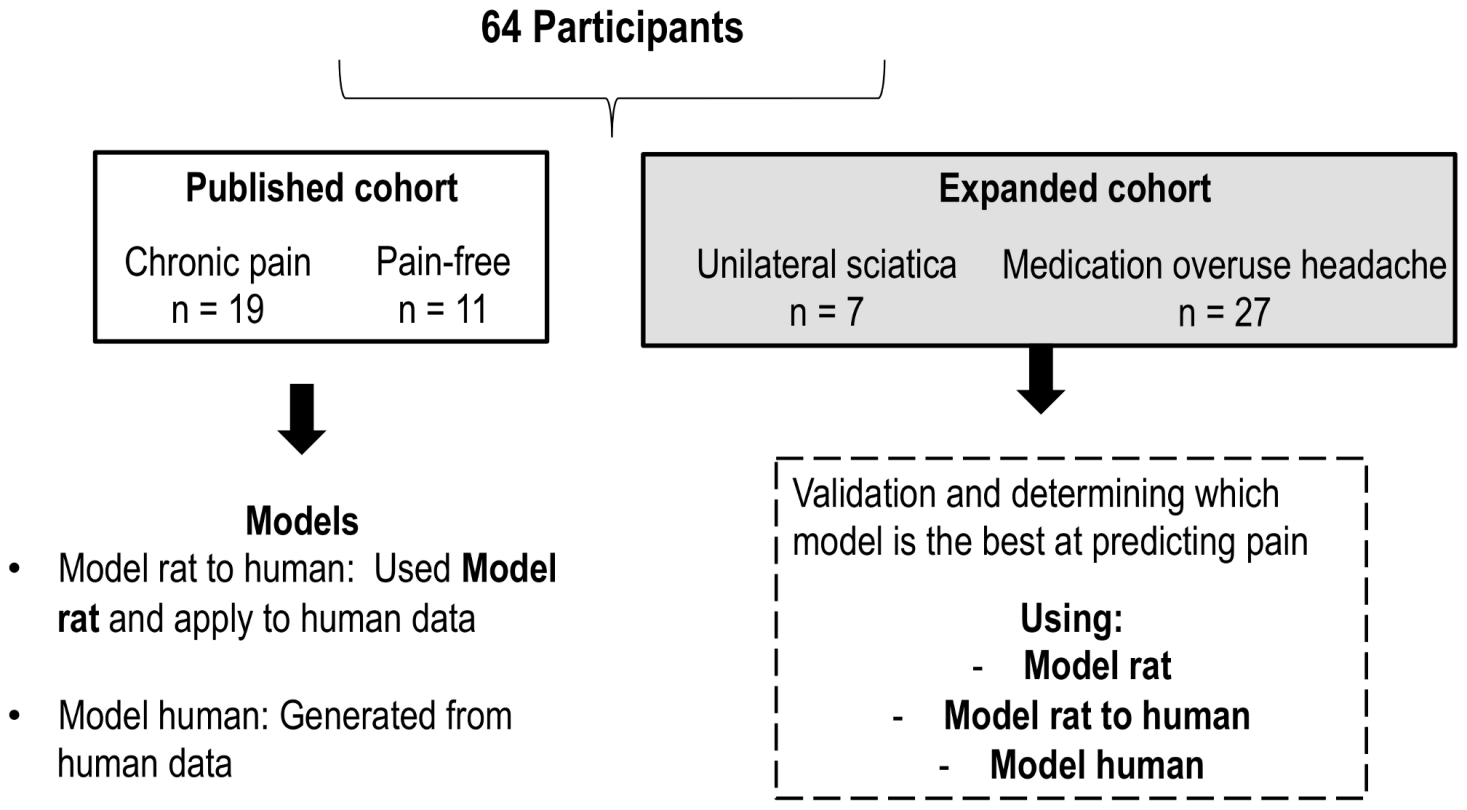
Overview of the generation and comparison of models generated from post graded CCI rats and humans (chronic pain and pain-free participants).

The overview of the modeling is summarized in Figure 3. The chronic pain patients in the published cohort [21] were the group considered to have pain therefore assigned as 1. The pain-free participants were considered to have no pain hence assigned as 0. The “Model human” was the refined model constructed from the collected outputs (Table 3) from the “published cohort” [21] with the use of the glm and stepAIC function.

**Table 3:** Summary of variables collected from humans in the periphery region.

Variables		Published Cohort		Expanded Cohort
		Chronic Pain	Pain-Free	Chronic Pain
**Non-stimulated**	**Cell count (^∧^7)**	1.1±0.09	1±0.12	1.2±0.07
	**Cells (pg/mL)**	0.9±0.2	1.1±0.2	12±3
**Pam3CSK4 stimulated (TLR2) IL-1β (pg/mL)**	**Minimum**	3.5±2.5	2.1±1.1	30±8.2
	**Maximum**	929.8±164.4	162.6±40.5	524.5±93.9
	**Slope**	155.4±33.1	31.60±14.2	65.75±15.4
	**Intercept**	677.5±148.3	93.4±19.3	361.7±69.6
**LPS stimulated (TLR4) IL-1β (pg/mL)**	**Minimum**	107.6±82	15.2±7.3	112.3±37.3
	**Maximum**	2231±202.2	2008±162	1975±145.3
	**Slope**	358.8±40.1	289.2±24.6	322.5±51.1
	**Intercept**	2065±207.6	1530±150.1	1930±239.2

Data are presented as mean ± SEM. Peripheral blood mononuclear cells (PBMC) were isolated from 64 participants and were stimulated with LPS (6 pg·ml^−1^ to 10 µg·ml^−1^) and with Pam3CSK4 (from 13 pg·ml^−1^ to 1 µg·ml^−1^) for 20 h.

#### Comparison of different models developed from rats and humans

“Model rat to human” used the output variables selected by the **Peripheral** subset obtained from rats (from **all** experimental groups) and applied to the clinical data obtained from the “published cohort” [21] (listed in Table 3). A ROC curve was generated from both models and the area under the curve was calculated. One-way ANOVA was used to compare the models generated from rats and from humans to predict the presence of pain.

### Validation of Models to Predict the Presence of Pain in a New Chronic Pain Cohort

The predict function in R was used to determine which of the constructed models (see Figure 3 and also listed below) is the best predictor of pain presence in the “expanded cohort” (all chronic pain participants). Participant with the predicted score between 0 to 0.5 was considered to have no pain and score above 0.5 was considered to have pain.

- Model rat: The output variables were from the **Peripheral** subset refined model obtained from rats and the data used in this model was also from rats.
- Model rat to human: “Model rat” was used however the rat data was replaced with human data (“published cohort”).
- Model human: Model generated from the published cohort.

### Statistical Analyses

Graphpad Prism version 6.0 for Windows (GraphPad Software, San Diego California USA, www.graphpad.com) was used for basic statistical analysis and correlation graphs unless otherwise stated. Data were tested for normality with the D’Agostino-Pearson omnibus normality test and when the data did not fit normal distribution a non-parametric test was chosen instead.

For study 1, data from the von Frey test were analyzed as the interpolated 50% threshold (absolute threshold) in log base 10 of stimulus intensity (monofilament stiffness in milligrams *10). The cell count data was normalized by log transformation. Differences between experimental groups in von Frey score and *in vitro* IL-1β post TLR agonist were analyzed using repeated measures two-way ANOVAs followed by Bonferroni *post hoc* test. The experimental groups differences on the day of cull and the cell count was analyzed with one-way ANOVA followed by Bonferroni *post hoc* test. For the other variables: plasma, basal IL-1β level, TLR agonist stimulated IL-1β curves (min, max, slope and intercept), TLR agonist stimulated IL-1β from spinal cord the experimental groups differences were calculated with Kruskal-Wallis one-way ANOVA.

For study 2, the age difference between the chronic pain participants (published and expanded cohort) and the pain-free participants was analyzed using one-way ANOVA. The daily morphine used and the duration of pain between the 2 chronic pain cohorts was analyzed using Mann-Whitney test. To determine the group differences between the new cohort of chronic pain patients and pain-free participants, previously published clinical data was used [21]. The concentration-response curve for the TLR2 agonist was assessed using a sigmoidal concentration response equation. For the TLR4 agonist concentration-response, curve a modified biphasic curve as described previously was used [21]. The *F-tests* were used to determine if the best fit curves with the selected parameters (E_max_, E_min_ and EC_50_) differed, thus reflecting group differences in the IL-1β expressed by PBMCs post TLR agonist stimulation.

For both studies, the concentration-response curves for the TLR2 and TLR4 agonists obtained from rats and humans were fitted by linear regression. The minimum, maximum, slope and intercept were calculated from the curves obtained from each rat group, chronic pain patients and pain-free participants. All significance was set at *P*<0.05.

## Results

### Study 1: Post Graded CCI Rat Model

#### Rats developed allodynia after CCI surgery

**Figure 4:**
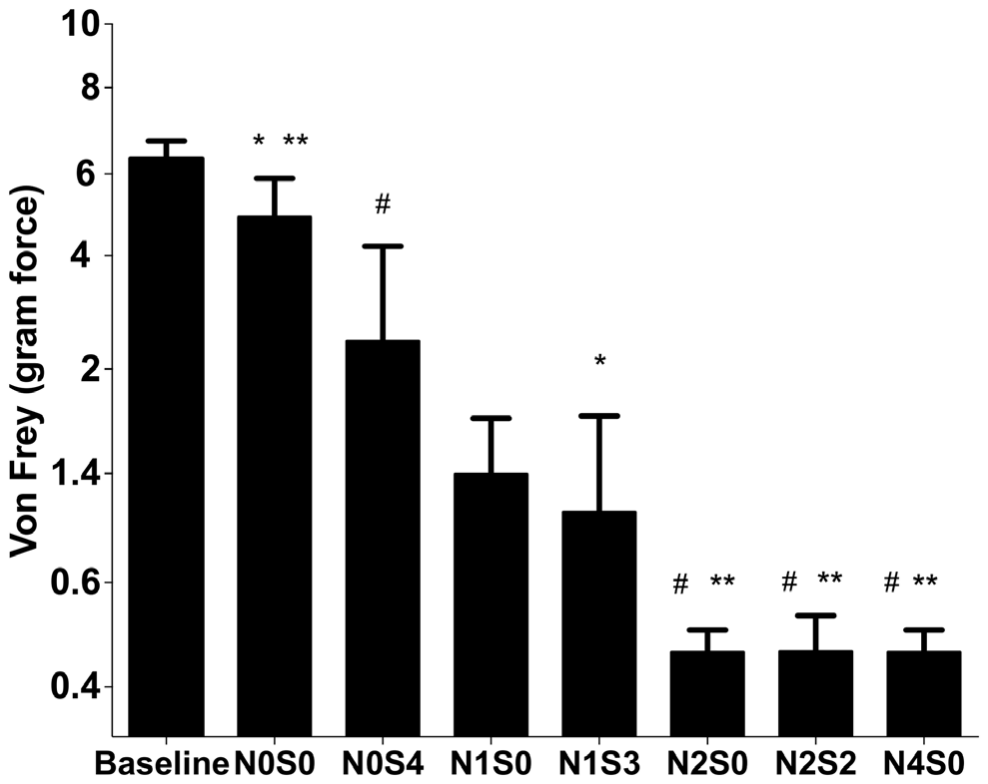
Allodynia quantification at day of cull (At least postoperative day 18). Graded neuropathy was induced by varying the number of chromic gut pieces ligating the nerve (N) and/or distributed in the subcutaneous (S) compartments. The treatment groups were N0S0, N0S4, N1S0, N1S3, N2S0, N2S2 and N4S0 (n = 6/group). At baseline all rats responded very similarly and was not included in the statistical analysis. A significant group effect was observed at day of cull (*P*<0.0001) and with some of the experimental groups (*P = 0.03, N0S0 vs. N1S3; **P = 0.0002, N0S0 vs. N2S0, N0S0 vs. N2S2, N0S0 vs. N4S0; #*P* = 0.02, N0S4 vs. N2S0, N0S4 vs. N2S2, N0S4 vs. N4S4). Error bars on graphs represent standard error of the mean and significance is set at *P*<0.05.

At baseline all rats had similar behavior scores revealed by one-way ANOVA (NS, P = 0.8) (Figure 4). After CCI surgery, two-way ANOVA revealed a significant effects of group (P<0.0001) and time (P<0.0001) (data not shown). On the day of cull (at least PO day 18 to PO day 27), two-way ANOVA revealed a significant group effect was observed (P<0.0001) and Bonferroni *post hoc* test showed N1S3 (P = 0.03), N2S0 (P = 0.0002), N2S2 (P = 0.0002) and N4S0 (P = 0.0002) having significantly greater allodynia when compared to sham N0S0. The following experimental groups: N2S0 (P = 0.02), N2S2 (P = 0.02) and N4S0 (P = 0.02) were also found to have significantly greater allodynia scores than N0S4 (no nerve ligatures) confirming the important component of nerve involvement (Figure 4).

#### IL-1β outputs (basal and stimulated) collected from rats in central and peripheral regions did not differ between experimental groups

The basal cell level (un-stimulated) of IL-1β expression was revealed by one-way ANOVA to be the same for all 7 experimental groups (p = 1) (see Table 1). In contrast to the previous human study, two-way ANOVA revealed there were no significant group effect between all 7 experimental groups post TLR2 (p = 0.9) or TLR4 (p = 0.1) agonist stimulation in the isolated PBMCs but a significant concentration effects was found for both TLR2 (p = 0.0002) and TLR4 (p = 0.001). No significant group effect was found by the Kruskal-Wallis one-way ANOVA for the lumbar spinal cord (Basal p = 0.8, Post TLR2 stimulated p = 0.2 and Post TLR4 stimulated p = 0.6) or from the supernatant (Basal p = 0.7, Post TLR2 stimulated p = 0.9 and Post TLR4 stimulated p = 0.9) showed in Table 2.

#### In all experimental groups Model A: Central outputs best predicted the presence of pain in rats

The panel of output variables that best predicted the presence of pain was from **Central** region (Table 4). The area under the ROC curve (ROC AUC) was 0.9 indicating a very good ability to determine the presence of pain. The IL-1β released by the basal spinal cord played a significant contribution in the model (P = 0.04). The order of best to worse models for the predictor of pain collected from the dataset and subsets are as follows: **All**>**Basal**>**TLR2** and **Peripheral**>**TLR4** (ROC AUC: 0.87>0.8>0.76>0. 61) (Additional information can be found in Table S1 in File S1). ANOVA analysis revealed the model generated from the **Complete** dataset did not differ with the model collected from the **Peripheral** (p = 0.09) but it was significantly different from the models generated from output variables collected from **Central** (p = 0.04), **TLR2** (p = 0.02), **TLR4** (p = 0.003) and **Basal** (p = 0.01).

**Table 4:** Best-fit logistic regression model results for the prediction of pain for rats post CCI.

ExperimentalGroups:	Dataset	Variables	Estimate	SE	*P*	Null deviance	df	Residual deviance	df	AUC	ANOVA
**All**	**Central**	TLR4 stimulated supernatant ofspinal cord	0.21	0.12	0.07.	39.90	34	23.57	29	0.9	0.004
		Non-stimulated supernatant ofspinal cord	−0.052	0.052	0.31						
		TLR4 stimulated spinal cord	0.099	0.052	0.058						
		TLR2 stimulated spinal cord	0.067	0.038	0.078						
		**Non-stimulated spinal cord**	−0.062	0.029	0.036						
**Neuronal and subcutaneous**	**Complete**	Peripheral non-stimulated plasma	0.78	0.60	0.19	31.76	23	16.76	18	0.9	–
		Peripheral non-stimulated cells	−0.31	0.23	0.17						
		Peripheral TLR4 stimulated min	0.10	0.059	0.085						
		Central TLR4 stimulatedspinal cord	0.070	0.056	0.21						
		Central non-stimulatedspinal cord	−0.073	0.049	0.14						
**Neuronal**	**Complete**	Peripheral non-stimulated cells	−1.23	0.77	0.11	23.05	20	14.51	18	0.86	–
		Peripheral TLR4 stimulatedintercept	0.43	0.31	0.17						

Notes: Significant variables are shown in bold. The discrimination probabilities (D, area under ROC curve) are presented in the table. One-way ANOVA was used to compare which subsets (Central/Peripheral/TLR2/TLR4 or Basal) when compare with all outputs is a better model. The residual deviance for the model includes predictor variables, whereas the null deviance for the model does not. SE, standard error.

#### In all experimental groups Model B: A combination of all outputs best predicted the severity of allodynia in rats

The combined output variables (include all regions and stimulations) that best predicted the severity of allodynia was from **All** outputs (see Table 5). The actual von Frey score was significantly correlated with the predicted von Frey score generated from a panel of output variables (adjusted R-square = 0.44, P = 0.0044). The following output variables played a significant contribution to the model: TLR2 stimulated PBMC responses (max (P = 0.01), min (P = 0.01) and intercept (P = 0.008), basal spinal cord (P = 0.007) and TLR2 stimulated spinal cord (P = 0.003) responses. The order of the other models generated from the different subsets are as follows: (from best to worse predictor of allodynia severity) **Central**>**TLR2**>**Peripheral>Basal>TLR4** (adjusted R-square: 0.32 (P = 0.0035) >0.17 (P = 0.05) >0.17 (P = 0.11) >0.1 (NS P = 0.07) >0.0081, (NS P = 0.37) (Additional information can be found in Table S2 in File S1). ANOVA analysis revealed the model generated from all outputs was significantly different from the **Peripheral** (p = 0.004), **TLR2** (p = 0.01), **TLR4** (p = 0.003) and the **Basal** (p = 0.01) subsets but it did not differ with **Central** (p = 0.09) collected outputs.

**Table 5:** Best-fit logistic regression model results for the prediction of the pain severity in rats post CCI in all experimental groups.

Dataset	Variables	Estimate	SE	*P*	Nulldeviance	df	Residualdeviance	df	AdjustedR-square:	P-value
**Complete**	Peripheral non-stimulated cell count	−1.99×10^−8^	1.30×10^−8^	0.14	5.42	34	2.15	24	0.44	0.0044
	Peripheral non-stimulated plasma	−1.22×10^−2^	8.84×10^−3^	0.18						
	Peripheral TLR4 stimulated min	1.56×10^−2^	8.69×10^−3^	0.086						
	Peripheral TLR4 stimulated intercept	−8.13×10^−3^	4.89×10^−3^	0.11						
	Peripheral TLR4 stimulated slope	−1.37×10^−2^	6.80×10^−3^	0.055						
	**Peripheral TLR2 stimulated max**	−5.29×10^−2^	1.91×10^−2^	0.011						
	**Peripheral TLR2 stimulated min**	−1.25×10^−1^	4.46×10^−2^	0.0099						
	**Peripheral TLR2 stimulated intercept**	1.19×10^−1^	4.11×10^−2^	0.0078						
	**Central TLR2 stimulated spinal cord**	−7.74×10^−3^	2.32×10^−3^	0.0028						
	**Central non- stimulated spinal cord**	6.36×10^−3^	2.15×10^−3^	0.0069						
	**Spinal cord**	0.0050	0.0024	0.043						

Notes: Significant variables are shown in bold. The residual deviance for the model includes predictor variables, whereas the null deviance for the model does not. SE, standard error.

#### In neuronal and subcutaneous experimental groups Model A: A combination of all outputs best predicted the presence of pain in rats

The panel of output variables that best predicted the presence of pain was from **All** output variables (Table 4). The ROC AUC was 0.9 and the order of best to worse predictor of pain of the other models collected from the other subsets are as follows: **Peripheral** and **Central**>**TLR2**> **TLR4**> **Basal** (ROC AUC: 0.88>0.77>0.64>0.58) (Table S1 in File S1). ANOVA analysis revealed the model generated from the **All** outputs variables did not differ with model collected from **Peripheral** (p = 0.2), **Central** (p = 0.2), **TLR2** (p = 0.2), **TLR4** (p = 0.05) or **Basal** (p = 0.1).

#### In neuronal and subcutaneous experimental groups Model B: TLR2 outputs best predicted the severity of allodynia in rats

The panel of output variables that best predicted the severity of allodynia was from **TLR2** IL-1β outputs (see Table 6). The von Frey score was significantly correlated with the variables from the **TLR2** IL-1β outputs (adjusted R-square: 0.37, P = 0.02). Within the output variables selected, the following outputs have significant contribution: TLR2 stimulated PBMC (max (P = 0.03), intercept (P = 0.04)) and TLR2 stimulated spinal cord (P = 0.01). The order of the other models collected from different dataset and subsets are as follows: (from best to worse predictor of allodynia severity) **Central >Peripheral>Basal**>**All>TLR4** (adjusted R-square: 0.34 (P = 0.02) >0.29 (P = 0.04) >0.23 (P = 0.04) >0.56 (NS P = 0.06)>−0.019 (NS P = 0.5) (Table S2 in File S1). ANOVA analysis revealed the model generated from **All** output variables was not significantly different from the **Peripheral** (p = 0.15), **Central** (p = 0.19), **TLR2** (p = 0.2), **TLR4** (p = 0.05) and **Basal** (p = 0.12) specific output variables.

**Table 6:** Best-fit logistic regression model results for the prediction of the pain severity in rats post CCI in neuronal and subcutaneous experimental groups.

Dataset	Variables	Estimate	SE	*P*	Nulldeviance	df	Residualdeviance	df	AdjustedR-square:	P-value	ANOVA
**TLR2 agonist** **stimulation only**	**Peripheral stimulated max**	−0.041	0.017	0.031	4.52	23	2.24	18	0.37	0.02	0.2
	Peripheral stimulated min	−0.073	0.058	0.23							
	**Peripheral stimulated intercept**	0.084	0.038	0.041							
	**Central stimulated spinal cord**	0.0091	0.0033	0.013							
	Central spinal cord supernatant	0.0046	0.0026	0.096.							
	**Central spinal cord**	0.010	0.0036	0.010							

Notes: Significant variables are shown in bold. One-way ANOVA was used to compare which subsets (Central/Peripheral/TLR2/TLR4 or Basal) when compare with all outputs is a better model. The residual deviance for the model includes predictor variables, whereas the null deviance for the model does not. SE, standard error.

#### In neuronal experimental groups Model A: A combination of all outputs best predicted the presence of pain in rats

The panel of output variables that best predicted the presence of pain was from the **All** outputs variables (Table 4). The ROC AUC was 0.86 indicating a very good ability to determine the presence of pain. The order of the best to worse predictor of allodynia of the other models collected from the other subsets are as follows: **Central** and **Basal**>**TLR2**>**Peripheral**>**TLR4** (ROC AUC: 0.76>0.66>0.63>0.6) (Table S1 in File S1). ANOVA analysis revealed the outputs generated from **Peripheral** (p = 0.009) and **TLR2** (p = 0.006) were significantly different from **All** output variables but not with **Central** (p = 0.16). The ANOVA could not be calculated for the model generated from **TLR4** output variables owing to incompatibility of the models.

#### In neuronal experimental groups Model B: A combination of all outputs best predicted the severity of allodynia in rats

The output variables that best predicted the severity of allodynia were from **All** output variables (see Table 7). The von Frey score was significantly correlated with the combined output variables (adjusted R-square: 0.67, P = 0.0048). Within the output variables selected the following outputs have significant contribution: non-stimulated cells (P = 0.001), TLR4 stimulated PBMC (max (P = 0.02) and slope (P = 0.03)) and TLR2 stimulated PBMC max (P = 0.02). The orders of the other models collected from the other subsets are as follows: (from best to worse predictor of allodynia severity) **Peripheral**>**Basal>Central>TLR2** (adjusted R-square: 0.62 (P = 0.004) >0.35 (P = 0.03) >0.16 (NS P = 0.08) >0.08 (NS P = 0.1) (Table S2 in File S1). Correlation between von Frey score and TLR4 only outputs was not obtained due to the refined model not being solved. ANOVA analysis revealed the model generated from **All** output variables was significantly different from the following outputs collected in **Central** (p = 0.0089), **TLR2** (p = 0.0063), **Basal** (p = 0.022) but it was not different from the outputs collected from **Peripheral** (p = 0.16).

**Table 7:** Best-fit logistic regression model results for the prediction of the pain severity in rats post CCI in neuronal experimental groups.

Dataset	Variables	Estimate	SE	*P*	Nulldeviance	df	Residualdeviance	df	AdjustedR-square:	P-value
**Complete**	Peripheral non-stimulated plasma	−0.013	0.007	0.088.	3.12	20	0.56	11	0.67	0.0048
	**Peripheral non-stimulated cells**	0.12	0.027	0.00094						
	**Peripheral TLR4 stimulated max**	−0.024	0.0089	0.022						
	**Peripheral TLR4 stimulated slope**	0.068	0.027	0.03						
	**Peripheral TLR2 stimulated max**	−0.037	0.014	0.022						
	Central TLR4 stimulated spinal cord supernatant	−0.023	0.015	0.15						
	Central non-stimulated spinal cord supernatant	0.013	0.0096	0.21						
	Central TLR2 stimulated spinal cord	−0.0045	0.003	0.16						
	Central non-stimulated spinal cord	0.0042	0.0021	0.065.						
	Peripheral plasma	−1.29×10^−2^	9.89×10^−3^	0.22						
	**Peripheral cell**	7.66×10^−2^	2.79×10^−2^	0.014						
	Central spinal cord supernatant	−1.267×10^−2^	6.041×10^−3^	0.052.						

Notes: Significant variables are shown in bold. The residual deviance for the model includes predictor variables, whereas the null deviance for the model does not. SE, standard error.

### Model C: Selected Peripheral Outputs can be Significantly Correlated with the IL-1β from the Lumbar Spinal Cord

#### All experimental groups

The refined model from the **Peripheral** subset correlated the best with the IL-1β released from the basal spinal cord culture supernatant (adjusted R-square: 0.26, P = 0.003) followed by TLR4 stimulated spinal cord culture supernatant (adjusted R-square: 0.22; P = 0.008) and lastly by TLR2 stimulated spinal cord culture supernatant (adjusted R-square: 0.08, P = 0.05). The same refined model was only correlated with the TLR2 stimulated spinal cord however it did not reach significance (adjusted R-square: 0.06, P = 0.24). Correlations could not be obtained from the TLR4 stimulated spinal cord or from the non-stimulated spinal cord.

#### Neuronal and subcutaneous experimental groups

**Figure 5:**
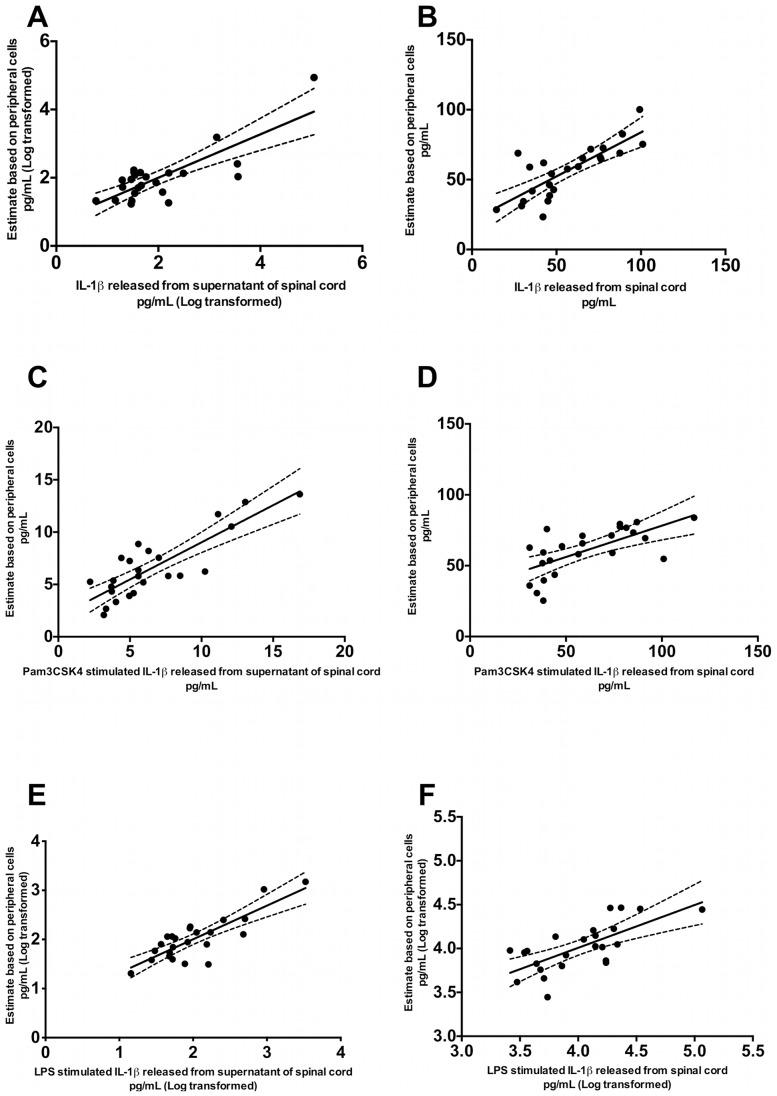
Rat spinal cord (basal, post TLR2 and TLR4 agonist stimulation) was positively correlated with periphery outputs. IL-1β level released from (A) basal spinal cord supernatant (*P* = 0.00048, adjusted R-square = 0.56) (B) basal spinal cord (*P* = 0.011, adjusted R-square = 0.47) (C) spinal cord supernatant (*P* = 0.01, adjusted R-square = 0.52) (D) spinal cord post Pam3CSK4 (*P* = 0.04, adjusted R-square = 0.29) (TLR2) stimulation at 100 µg/mL (E) spinal cord supernatant (*P* = 0.02, adjusted R-square = 0.48) (F) spinal cord (*P* = 0.09, adjusted R-square = 0.26) post LPS (TLR4) stimulation at 100 µg/mL was found to be significantly correlated with the estimated values predicted from peripheral tissue outputs in rats from Neuronal and subcutaneous. Pearson correlation was used and data shown in panel (A, E and F) have been log transformed and linear regression with 95% confidence interval curves are shown on the graph.

The refined model from the **Peripheral** subset best correlated with the basal spinal cord culture supernatant (adjusted R-square: 0.56, P = 0.0005) (Figure 5A) followed by TLR2 stimulated spinal cord culture supernatant (adjusted R-square: 0.52; P = 0.01) (Figure 5C) and lastly by TLR4 stimulated spinal cord culture supernatant (adjusted R–square: 0.48, P = 0.02) (Figure 5E). The refined model generated from output variables collected from **Peripheral** location best correlated with the non-stimulated spinal cord culture supernatant (adjusted R-value: 0.56, P = 0.0005) (Figure 5B) followed by TLR2 stimulated spinal cord culture supernatant (adjusted R-square: 0.52; P = 0.01) (Figure 5D) and lastly TLR4 stimulated spinal cord culture supernatant (adjusted R–square: 0.48, P = 0.02) (Figure 5F).

### Study 2: Chronic Pain and Pain-free Participants

#### Human participant demographic data

Basic demographics are listed in Table 8. In the published cohort [21] there are nineteen chronic pain patients (13 female, 6 male, (min-max) 33–65 years old; mean age 52), and eleven pain-free participants (7 female, 4 male; 36–61 years old; mean age 51). In the expanded cohort there are thirty-four chronic pain patients (25 female, 9 male, 23–64 years old, mean age 46). Additional information on the pain diagnosis of chronic pain patients can be found in Table 9. The average duration of pain in the published cohort was 7 years (min-max; 1–28) and for the expanded cohort was 21 years (min – max, 3–55). The mean daily dose (oral morphine equivalent) taken by the published cohort was (mean ± SEM) 49±13 mg and for the expanded cohort was 13±3 mg.

**Table 8:** Demographic summary.

	Published Cohort		*Expanded Cohort*	
	Chronic pain(n = 19)	Pain-free(n = 11)	Chronic Pain(n = 34)	*P*
**Gender (M/F)**	6/13	4/7	9/25	–
**Age (Years)**	52 (33–65)	51 (36–61)	46 (23–64)	0.17
**Oral morphine equivalent dose** **(per day) (mg)**	49±13	–	13±3	0.44
**Duration of chronic pain (Years)**	7±2	–	21±2	<0.0001

Data were collected from medical and family history. Data are expressed as mean ± S.E.M except age is expressed as mean ± min–max. One-way ANOVA was used to determine the age difference and the non-parametric Mann-Whitney test was used to daily morphine dose and duration of chronic pain between the chronic pain groups (P-values shown).

**Table 9:** Primary diagnoses and medications of chronic pain patients (n = 53).

Diagnosis	Number (%)		
	PublishedCohort		ExpandedCohort
Chronic back or shoulder or leg pain	26.3		–
Fibromyalgia	10.6		–
Sciatica	10.6		20.5
Osteoarthritis	31.5		–
Medication-overuse headache	–		79.5
Others	21*		–
**Medications**			
Opioids	58	79.4	
On medications other than opioids:	32	8.8	
Not on medications	10	11.8	

* Other pain diagnosis include: complex regional pain syndrome (n = 1), atypical trigeminal neuralgia (n = 1), neuropathic Pain syndrome (n = 1) and non cardiac chest pain (n = 1).

#### Increased TLR responsiveness was observed in the isolated PBMCs collected from the expanded cohort compared with pain-free participants

**Figure 6:**
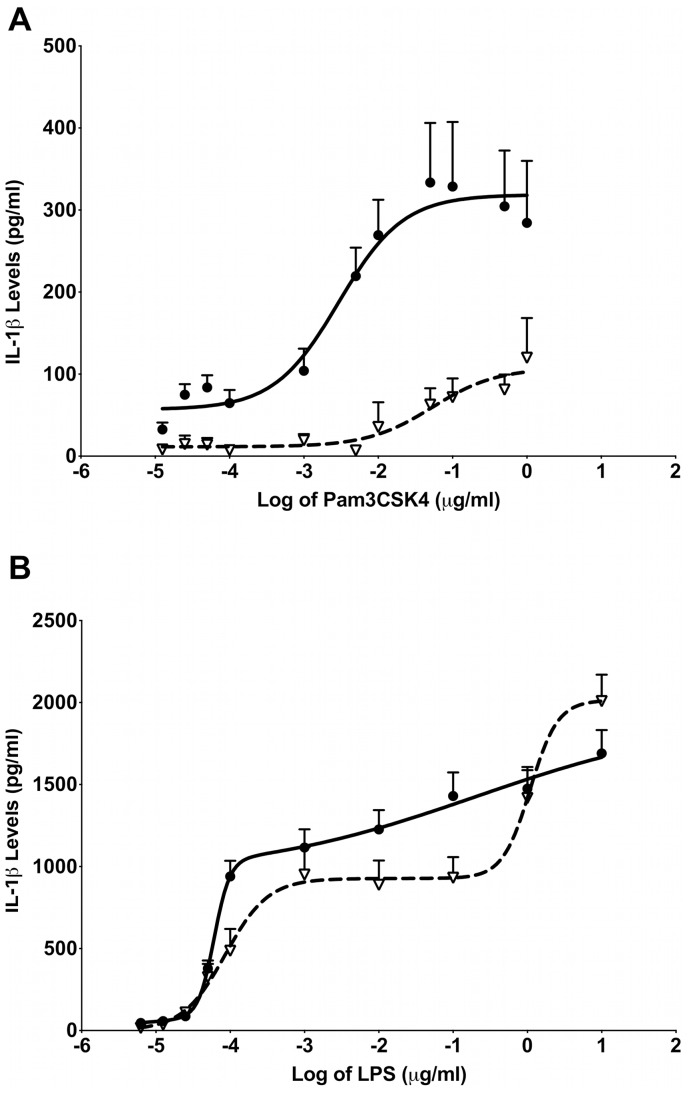
TLR agonist stimulation caused significant group differences in the release of IL-1β in chronic pain patients and pain-free participants. Isolated white cells obtained from new chronic pain patients (closed circle) and pain-free controls from previous study (open triangle) were stimulated with a range of (A) Pam3CSK4 (TLR2) concentrations (13 pg·ml^−1^ to 1 µg·ml^−1^) and (B) LPS (TLR4) concentrations (6 pg·ml^−1^ to 10 µg·ml^−1^) to generate the response curves and resulted in significant group differences (Pam3CSK4; *P*<0.0001 and LPS, *P* = 0.004). Error bars on graphs represent standard error of the mean.

The TLR2 agonist Pam3CSK4 induced significant concentration-dependent increases in IL-1β release in the isolated PBMCs collected from the chronic pain patients (expanded cohort) when compared with pain-free participants from the previously published cohort [21]. The clear separation between the two groups resulted in an overall significant group effect in response to Pam3CSK4 (*F*_3, 452_ = 13, *P<*0.0001; see Figure 6A).

The TLR4 agonist LPS induced elevations in IL-1β in the isolated PBMCs collected from the chronic pain patients in the expanded cohort and in pain-free participants from the published cohort [21]. There was a significant group difference (*F*_1, 385_ = 5, *P<*0.03; see Figure 6B).

### Which Model is Best at Predicting Pain Presence

#### Models generated from peripheral derived models from both rats and humans have a good ability to predict presence of pain in human

**Figure 7:**
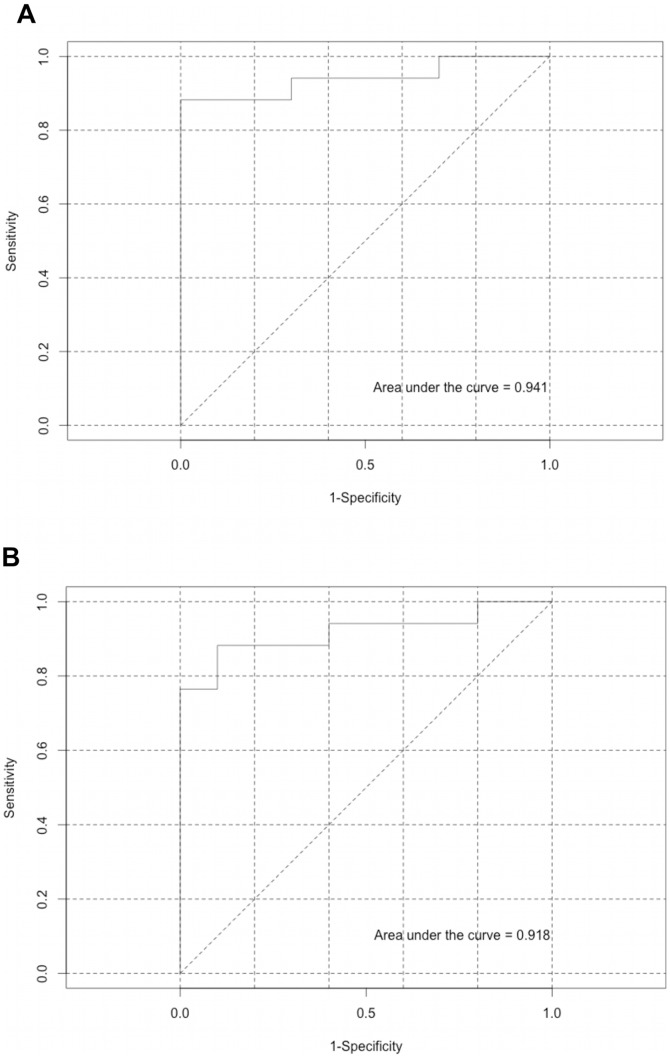
Representation of ROC curves for the detection of pain presence. Models generated from (A) rat data and (B) human data obtained from peripheral collected output variables.

The ROC AUC generated from the “Model rat to human” was 0.94 indicating a very good ability to determine the presence of pain in humans (published cohort; Figure 7A). Likewise, the “Model human” had an ROC AUC of 0.92 (Figure 7B) (Additional information of the model can be seen in Table S3 in File S1) also indicating a very good ability to detect the presence of pain. ANOVA analysis revealed the two models were found to be not significantly different (Table S3 in File S1).

#### Model rat was found to predict presence of pain accurately in a cohort of chronic pain participants

**Figure 8:**
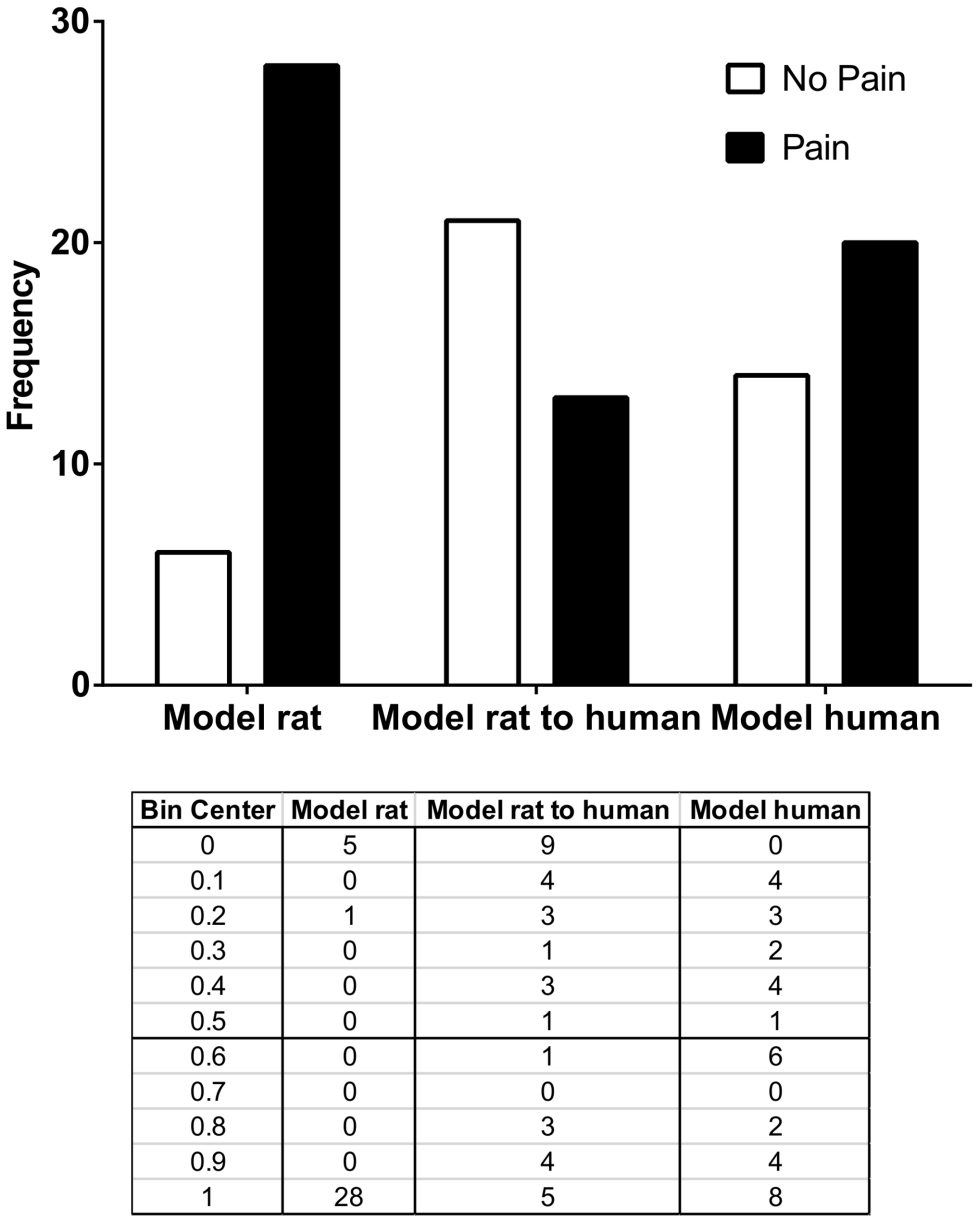
Representation of the constructed models Model rat, Model rat to human and Model human that predicted the presence of pain in the expanded cohort. Bin center 0 represents no pain and 1 represents pain.

All participants in the expanded cohort should be 1 (pain) however according to all models some participants were predicted to have no pain (Figure 8). The number of participants predicted to have no pain/pain (from best to worst predictors) by “Model rat” was 6/28, for “Model rat to human” was 21/13 and for Model human” was 14/20.

## Discussion

Despite the wealth of pre-clinical evidence implicating TLR-mediated neuroinflammation and chronic pain [9]–[11], [26], the relevance of neuroinflammation and TLR signaling in human pain conditions is lacking largely due to the inaccessibility of the CNS [3]. However, we have recently published functional evidence of this relationship by demonstrating that low dose intravenous endotoxin (LPS; TLR4 activator) markedly enhanced the flare, hyperalgesia and allodynia responses to intradermal capsaicin in healthy volunteers [27]. Despite this important finding, this model is not practical as a pain biomarker in large patient populations. Hence more practical biomarkers of the neuroimmune activation status in chronic pain are needed. In this study we have two major findings relevant to this aim. The first is that we have replicated our earlier conclusion that *in vitro* PMBC stimulation to be a biomarker of the pain state in two distinct patient populations. Sciatica was selected as it has high face validity to the CCI model used in our animal experiments. Medication overuse headache was selected in contrast as there is no evidence of peripheral pathology in this condition, yet we have hypothesized that opioid-induced glial activation is a major contributing pathology [28]. However cross-sectional studies demonstrating correlation cannot demonstrate a causal relationship. Hence our second major finding is that in a prospective graded rat model we have shown that immune activation in peripheral blood and in spinal cord were related to the pain state in a “dose”-related manner supporting a causal relationship. There are two main implications of these findings. Firstly, this confirms the likely role of TLR signaling in human chronic pain, providing support to the search for inhibitors of these systems as potential new treatment for pain. Secondly, excitingly, as the sensitivity is measured in the readily accessible tissue of peripheral blood, these assessments have the potential to act as biomarkers.

Biomarkers of pain have several potential clinical utilities. One important potential role would be to support patient stratification for enriched clinical trials or for monitoring the response to intervention [29], [30], making such trials more sensitive to interventions and more meaningful. Another potential role is in patients who cannot communicate well, e.g. children, patients with cognitive impairment, or where cultural and language barriers prevent meaningful evaluation or comparison between populations [31].

### The Superior Discrimination of Stimulated vs. Basal Immune Responses

Our data have indicated that the innate immune responses following TLR2 and TLR4 stimulation are both linked to the presence of chronic pain. However, in humans, no group difference was detected from the basal (i.e. unstimulated) cell activity. In contrast, under stimulation conditions, discrimination between the pain/no pain groups was possible. In rats, a combination of basal output responses could identify the pain presence and the severity of allodynia. However, more sensitive and specific findings were obtained with the addition of the stimulated response. Therefore, it is important to not only examine the basal but also dynamic stimulation, as it allows for integrated ligand/receptor interaction, receptor to intracellular signaling, transcriptional to translational modification including genetic variability and epigenetic contributions. By only examining the basal response the above integration is lost and the elicit response has proven to be important for the discovery of potential pain biomarkers.

### How has the Rodent Work Added to Our Previous Findings?

Firstly, although cross-sectional studies in patients are easy to perform, they suffer from the inherent weakness of potential selection bias, and are hence at best hypothesis generating. Prospective longitudinal studies (from pre to post injury) in humans with neuropathic pain are difficult, as studying the patients before the onset of injury is probably only possible in post-surgical neuropathic pain, which is only one facet of the condition. Since only a minority of patients experience such complications, such studies are difficult because of very large sample sizes required potentially and complicated by the pathology for which the surgery is indicated (eg the altered immune milieu in cancer). Hence there are several advantages of studying the neuroimmune processes involved in pain in animals. Firstly, a study published recently found putative pain biomarkers from blood-based RNA transcriptome using the same preclinical graded model of pain [32]. Many of the genes identified encode for proteins that have a recognized role in nociceptive and immune signaling thus providing validation for the use of this model. Secondly, animals can be studied in a prospective manner in disease-free groups of little heterogeneity. More importantly, CNS tissue can be accessed directly. In this study we have shown that peripheral blood TLR signaling sensitivity performed in a similarly predictive manner to that from CNS-tissue sensitivity, providing construct validation to our findings in humans. However, the discrimination of pain states and degree of allodynia in rats was not possible on single derived parameters from either TLR 2 or 4 stimulation, unlike in our result in humans.

### IL-1β Expression from Lumbar Spinal Cord Predicted by Peripheral Combination Outputs

The output variables collected from peripheral sites proved to be informative in predicting central responses. Here we demonstrated that IL-1β expression from the rat’s lumbar spinal cord was related to peripheral immune cell responsivity. Even though the adjusted R-square was low, the significance of the correlation should not be dismissed. The findings imply that samples collected from the readily accessible peripheral circulation may provide information as to how the CNS is responding. Thus a peripheral marker for proinflammatory glial reactivity may be achieved without the need to access central tissues. It is speculated that the peripheral variables collected from chronic pain sufferers could also predict the IL-1β expression in the central region. Further studies are required to validate this exciting hypothesis.

### The Usefulness of Neuronal and Subcutaneous Experimental Groups

The graded CCI model allows for a better pain prediction when chromic gut is placed both around the sciatic nerve and in the subcutaneous space. This is supported by ROC AUC being 0.9 indicating the high accuracy to predict pain presence. The peripheral outputs collected from the neuronal and subcutaneous experimental groups could also be significantly correlated with central tissue IL-1β outputs. Peripheral immune cells are known to play a pivotal role in the establishment of chronic pain by infiltration into central sites [33] and interact with glia causing the release of pro-inflammatory mediators [34]. The neuronal and subcutaneous experimental group is recommended for the understanding of chronic pain as it better mimics the clinical heterogeneous phenotype rather than the standard binomial model of CCI [23].

### TLR Responsiveness in the New Cohort of Chronic Pain Patients

The level of IL-1β released from the new cohort of chronic pain patients was not as high as previously published chronic pain patients [21] and could be attributed the fact that the underlying pain is very different in medication-overuse headache and sciatica patients compared with the heterogeneous chronic pain population employed previously. The involvement of TLR signaling with medication-overuse headache patients is currently unknown and has not yet been reported. It should be noted the mean daily morphine equivalent dose of opioids in the new cohort was significantly less even though pain was experienced longer compared with the published cohort.

From the previous study and confirmed in the current study, chronic pain patients have greater TLR-induced IL-1β release from PBMCs than pain-free participants. The mechanistic cause of the PMBC phenotype that resulted in elevated TLR-induced IL-1β release is currently unknown. It is speculated that in chronic pain patients PBMCs are primed by previous exposure with DAMPs and hence following subsequent exposure to a TLR stimulus will produce an exaggerated response (increased in IL-1β release).

The technique of stimulating acutely isolated human PBMCs with TLR agonists and measuring cytokines has been previously used to examine innate immune function in patients. With the use of this cell culture technique, differences in cell reactivity have been detected between healthy controls and patients with the following conditions: surgery [35], rheumatoid arthritis [36], immunosuppression [37] and chronic fatigue syndrome [38]. This supports the usefulness of this technique to reflect the dysregulation of the immune function via the assessment of TLR signaling efficiency. Further research is required to identify which populations of cells in chronic pain patients are responsible for this increased IL-1β production. Using this acute isolation and culturing approach, little time is provided to allow the cells to differentiate away from their in vivo phenotype, thus providing results as close to the in vivo setting as experimentally possible. We speculate the underlying mechanisms lie in the intracellular TLR signaling as surface TLR expression does not always correspond to the PBMCs’ output as reported previously [37].

### Models Generated from Rat Data can Predict the Presence of Pain in Humans

The model generated from the rat was found to have very good prediction ability for the pain presence in the new cohort of chronic pain patients. This indicates the findings from rats could be translated to humans. The ability of the rat model to be able to predict the presence of pain could be attributed to more output variables being selected. Despite the high accuracy in the prediction of pain in chronic pain participant we do not believe that the current biomarker is as yet a diagnostic for pain. However, it does provide further evidence in humans of the importance of peripheral and central reactivity and that this biomarker approach might be useful in assessing the response to selecting intervention for evaluation and reflecting the response for novel treatments that target the TLR pathways. For it to be a clinically usable biomarker it will need to fulfill additional criteria such as discrimination between other disease states and sensitivity to treatment responses.

### Limitations of Current Study

There are several limitations in this study. Only 1 pro-inflammatory cytokine was examined in the study as we were testing a simplified system. As we were validating a previous finding here we did not wish to introduce new pain mediators. Secondly, this study only undertook collection of rat biological samples on the day of cull. It would be informative to conduct a longitudinal study to examine the time sequence and evolution in the sensitivity of the output variables to predict pain. Lastly, the same pain-free participants were used as a comparison to investigate whether increased TLR responsiveness was also observed with the expanded subject cohort and a larger control group would be useful.

### Conclusions

In summary, our study is novel, in that the data-driven approach was able to accurately predict pain presence and degree of allodynia in rats after graded CCI surgery. The peripherally derived model identified from rats could also be applied to humans and allowed the prediction of pain presence with accuracy. In addition, IL-1β levels in the central tissue could be predicted by the peripheral outputs obtained from the rats. Collectively, these results provide further evidence of the potential of peripheral cells in being a source of potential pain biomarkers that can be easily accessed and that supporting the role of TLR pathways in playing a vital role in the understanding of chronic pain.

## Supporting Information

File S1 **Contains: Table S1.** Best-fit logistic regression model results for the prediction of pain for rats post CCI. **Table S2.** Best-fit logistic regression model results for the prediction of the pain severity in rats post CCI. **Table S3.** Best-fit logistic regression model from rats (Peripheral only) and from humans to predict the presence of pain in chronic pain patients.
(DOCX)
