# Supplementary material for: TLR 2 and 4 Responsiveness from Isolated Peripheral Blood Mononuclear Cells from Rats and Humans as Potential Chronic Pain Biomarkers
Source: PLoS One. 2013 Oct 30;8(10):e77799. doi: 10.1371/journal.pone.0077799 (PMC3813723; doi:10.1371/journal.pone.0077799)
Supplement: File S1 — Contains: Table S1. Best-fit logistic regression model results for the prediction of pain for rats post CCI. Table S2. Best-fit logistic regression model results for the prediction of the pain severity in rats post CCI. Table S3. Best-fit logistic regression model from rats (Peripheral only) and from humans to predict the presence of pain in chronic pain patients. (DOCX) [file pone.0077799.s001.docx]

**Supporting documents for “TLR 2 and 4 responsiveness from isolated peripheral blood mononuclear cells from rats and humans as potential chronic pain biomarkers”**

**Table S1. Best-fit logistic regression model results for the prediction of pain for rats post CCI.**

|  | **Dataset** | **Variables** | **Estimate** | **SE** | ***P*** | **Null deviance** | **df** | **Residual deviance** | **Df** | **AUC** | **ANOVA** |
| --- | --- | --- | --- | --- | --- | --- | --- | --- | --- | --- | --- |
| **All data** | **Complete** | **Peripheral non-stimulated plasma** | 0.91 | 0.41 | 0.027 * | 39.90 | 34 | 26.27 | 32 | 0.87 | - |
|  |  | Central TLR4 stimulated spinal cord | 0.063 | 0.032 | 0.050 |  |  |  |  |  |  |
|  | **Peripheral** | Non-stimulated cell count | 9.17e-08 | 1.02e-07 | 0.37 | 49.57 | 40 | 40.65 | 33 | 0.76 | 0.09 |
|  |  | Non-stimulated plasma | 5.11e-01 | 2.97e-01 | 0.086 . |  |  |  |  |  |  |
|  |  | TLR4 stimulated max | 5.88e-02 | 9.18e-02 | 0.52 |  |  |  |  |  |  |
|  |  | TLR4 stimulated intercept | - 2.21e-02 | 1.11e-01 | 0.84 |  |  |  |  |  |  |
|  |  | TLR4 stimulated slope | -9.43e-02 | 1.91e-01 | 0.62 |  |  |  |  |  |  |
|  |  | TLR2 stimulated max | -7.38e-02 | 6.7e-02 | 0.29 |  |  |  |  |  |  |
|  |  | TLR2 stimulated min | -1.58e-03 | 2.79e-01 | 0.1 |  |  |  |  |  |  |
|  | **Central** | TLR4 stimulated supernatant of spinal cord | 0.21 | 0.12 | 0.07. | 39.90 | 34 | 23.57 | 29 | 0.9 | 0.004 |
|  |  | Non-stimulated supernatant of spinal cord | -0.052 | 0.052 | 0.31 |  |  |  |  |  |  |
|  |  | TLR4 stimulated spinal cord | 0.099 | 0.052 | 0.058 |  |  |  |  |  |  |
|  |  | TLR2 stimulated spinal cord | 0.067 | 0.038 | 0.078 |  |  |  |  |  |  |
|  |  | **Non-stimulated spinal cord** | -0.062 | 0.029 | 0.036 |  |  |  |  |  |  |
|  | **TLR2 stimulated only (peripheral & central)** | Peripheral stimulated max | 0.25 | 0.14 | 0.085 | 39.90 | 34 | 31.76 | 30 | 0.76 | 0.02 |
|  |  | Peripheral stimulated min | 0.55 | 0.36 | 0.13 |  |  |  |  |  |  |
|  |  | Peripheral stimulated intercept | -0.53 | 0.34 | 0.12 |  |  |  |  |  |  |
|  |  | Central stimulated spinal cord | 0.039 | 0.022 | 0.074 . |  |  |  |  |  |  |
|  | **TLR4 stimulated only (peripheral & central)** | Central stimulated spinal cord | 0.042 | 0.024 | 0.087 . | 39.9 | 34 | 35.88 | 33 | 0.61 | 0.003 |
|  | **Non-stimulated (peripheral & central)** | **Plasma** | 0.83 | 0.39 | 0.034 | 39.9 | 34 | 31.74 | 33 | 0.8 | 0.01 |
| **Neuronal and subcutaneous:** | **Complete** | Peripheral non-stimulated plasma | 0.78 | 0.60 | 0.19 | 31.76 | 23 | 16.76 | 18 | 0.9 | - |
|  |  | Peripheral non-stimulated cells | -0.31 | 0.23 | 0.17 |  |  |  |  |  |  |
|  |  | Peripheral TLR4 stimulated min | 0.10 | 0.059 | 0.085 |  |  |  |  |  |  |
|  |  | Central TLR4 stimulated spinal cord | 0.070 | 0.056 | 0.21 |  |  |  |  |  |  |
|  |  | Central non-stimulated spinal cord | -0.073 | 0.049 | 0.14 |  |  |  |  |  |  |
|  | **Periphery** | Non-stimulated plasma | 0.53 | 0.45 | 0.24 | 40.38 | 29 | 22.89 | 19 | 0.88 | 0.2 |
|  |  | Non-stimulated cells | -0.8 | 0.49 | 0.10 |  |  |  |  |  |  |
|  |  | **TLR4 stimulated max** | 0.77 | 0.36 | 0.034 |  |  |  |  |  |  |
|  |  | **TLR4 stimulated min** | 1.4 | 0.70 | 0.045 |  |  |  |  |  |  |
|  |  | **TLR4 stimulated intercept** | -1.94 | 0.93 | 0.037 |  |  |  |  |  |  |
|  |  | **TLR4 stimulated slope** | -1.86 | 0.90 | 0.038 |  |  |  |  |  |  |
|  |  | TLR2 stimulated max | 0.76 | 0.62 | 0.22 |  |  |  |  |  |  |
|  |  | TLR2 stimulated min | 1.3 | 1.01 | 0.20 |  |  |  |  |  |  |
|  |  | TLR2 stimulated intercept | -1.76 | 1.47 | 0.23 |  |  |  |  |  |  |
|  |  | TLR2 stimulated slope | -1.34 | 1.037 | 0.20 |  |  |  |  |  |  |
|  | **Central** | TLR2 stimulated supernatant of spinal cord | 0.54 | 0.34 | 0.11 | 31.76 | 23 | 18.03 | 18 | 0.88 | 0.2 |
|  |  | Non-stimulated supernatant of spinal cord | -0.16 | 0.10 | 0.11 |  |  |  |  |  |  |
|  |  | TLR4 stimulated spinal cord | 0.12 | 0.069 | 0.093 |  |  |  |  |  |  |
|  |  | TLR2 stimulated spinal cord | 0.053 | 0.036 | 0.14 |  |  |  |  |  |  |
|  |  | **Non-stimulated spinal cord** | -0.075 | 0.036 | 0.039 |  |  |  |  |  |  |
|  | **TLR2 stimulated only (peripheral & central)** | Peripheral stimulated slope | 0.4 | 0.27 | 0.13 | 31.76 | 23 | 26.59 | 21 | 0.77 | 0.2 |
|  |  | Central stimulated spinal cord | 0.032 | 0.022 | 0.16 |  |  |  |  |  |  |
|  | **TLR4 stimulated only (peripheral & central)** | Central stimulated spinal cord | 0.039 | 0.026 | 0.14 | 31.76 | 23 | 28.70 | 22 | 0.64 | 0.05 |
|  | **Basal** | Peripheral plasma | 0.55 | 0.39 | 0.16 | 31.76 | 23 | 27.40 | 22 | 0.58 | 0.1 |
| **Neuronal Only:** | **Complete** | Peripheral non-stimulated cells | -1.23 | 0.77 | 0.11 | 23.05 | 20 | 14.51 | 18 | 0.86 | - |
|  |  | Peripheral TLR4 stimulated intercept | 0.43 | 0.31 | 0.17 |  |  |  |  |  |  |
|  | **Periphery** | Non-stimulated cell count | 8.11e-08 | 1.48e-07 | 0.58 | 26.40 | 22 | 26.08 | 21 | 0.63 | 0.009 |
|  | **Central** | TLR2 stimulated supernatant of spinal cord | 0.22 | 0.22 | 0.32 | 23.05 | 20 | 21.56 | 19 | 0.76 | 0.16 |
|  | **TLR2 agonist stimulation only** | Peripheral stimulated max | 0.19 | 0.19 | 0.33 | 23.05 | 20 | 21.77 | 19 | 0.66 | 0.0062 |
|  | **TLR4 agonist stimulation only** | Central stimulated spinal cord | 0.038 | 0.032 | 0.24 | 23.05 | 20 | 21.27 | 19 | 0.60 | N/A |
|  | **Basal** | Peripheral cell count | 3.05e-07 | 2.6e-07 | 0.24 | 23.05 | 20 | 21.13 | 19 | 0.76 | 0.022 |

Notes: Significant variables are shown in bold. The residual deviance for the model includes predictor variables, whereas the null deviance for the model does not. The discrimination probabilities (D, area under ROC curve) are presented in the table. One-way ANOVA was used to compare which sub groups (central /peripheral/TLR2 agonist stimulation/ TLR4 agonist stimulation or Basal) when compare with all data set is a better model. The residual deviance for the model includes predictor variables, whereas the null deviance for the model does not. SE, standard error.

**Table S2. Best-fit logistic regression model results for the prediction of the pain severity in rats post CCI.**

|  | **Dataset** | **Variables** | **Estimate** | **SE** | ***P*** | **Null deviance** | **df** | **Residual deviance** | **Df** | **Adjusted R square:** | **P-value** | **ANOVA** |
| --- | --- | --- | --- | --- | --- | --- | --- | --- | --- | --- | --- | --- |
| **All data** | **Complete** | Peripheral non-stimulated cell count | -1.99e-08 | 1.30e-08 | 0.14 | 5.42 | 34 | 2.15 | 24 | 0.44 | 0.0044 |  |
|  |  | Peripheral non-stimulated plasma | -1.22e-02 | 8.84e-03 | 0.18 |  |  |  |  |  |  |  |
|  |  | Peripheral TLR4 stimulated min | 1.56e-02. | 8.69e-03 | 0.086 |  |  |  |  |  |  |  |
|  |  | Peripheral TLR4 stimulated intercept | -8.13e-03 | 4.89e-03 | 0.11 |  |  |  |  |  |  |  |
|  |  | Peripheral TLR4 stimulated slope | -1.37e-02 | 6.80e-03 | 0.055 |  |  |  |  |  |  |  |
|  |  | **Peripheral TLR2 stimulated max** | -5.29e-02 | 1.91e-02 | 0.011 |  |  |  |  |  |  |  |
|  |  | **Peripheral TLR2 stimulated min** | -1.25e-01 | 4.46e-02 | 0.0099 |  |  |  |  |  |  |  |
|  |  | **Peripheral TLR2 stimulated intercept** | 1.19e-01 | 4.11e-02 | 0.0078 |  |  |  |  |  |  |  |
|  |  | **Central TLR2 stimulated spinal cord** | -7.74e-03 | 2.32e-03 | 0.0028 |  |  |  |  |  |  |  |
|  |  | **Central non- stimulated spinal cord** | 6.36e-03 | 2.15e-03 | 0.0069 |  |  |  |  |  |  |  |
|  | **Periphery** | **Non-stimulated cell count** | -3.19e-08 | 1.49e-08 | 0.040 | 6.47 | 40 | 4.30 | 32 | 0.17 | 0.11 | 0.0036 |
|  |  | Non-stimulated plasma | -1.36e-02 | 1.05e-02 | 0.20 |  |  |  |  |  |  |  |
|  |  | TLR4 stimulated max | -5.58e-03 | 3.24e-03 | 0.095 . |  |  |  |  |  |  |  |
|  |  | TLR4 stimulated min | 1.04e-02 | 6.91e-03 | 0.14 |  |  |  |  |  |  |  |
|  |  | TLR4 stimulated intercept | 5.87e-03 | 3.65e-03 | 0.12 |  |  |  |  |  |  |  |
|  |  | **TLR2 stimulated max** | -5.32e-02 | 2.33e-02 | 0.029 |  |  |  |  |  |  |  |
|  |  | **TLR2 stimulated min** | -1.36e-01 | 4.94e-02 | 0.0095 |  |  |  |  |  |  |  |
|  |  | **TLR2 stimulated intercept** | 1.24e-01 | 4.93e-02 | 0.017 |  |  |  |  |  |  |  |
|  | **Central** | **TLR4 stimulated spinal cord supernatant** | -0.018 | 0.0083 | 0.037 | 5.42 | 34 | 3.26 | 30 | 0.32 | 0.0035 | 0.093 |
|  |  | **Non-stimulated spinal cord supernatant** | 0.0049 | 0.0023 | 0.043 |  |  |  |  |  |  |  |
|  |  | **TLR2 stimulated spinal cord** | -0.0076 | 0.0024 | 0.0041 |  |  |  |  |  |  |  |
|  |  | **Non-stimulated spinal cord** | 0.0079 | 0.0023 | 0.0016 |  |  |  |  |  |  |  |
|  | **TLR2 agonist stimulation only** | Peripheral stimulated max | -0.030 | 0.016 | 0.077 | 5.42 | 34 | 3.96 | 30 | 0.17 | 0.05 | 0.015 |
|  |  | **Peripheral stimulated min** | -0.12 | 0.045 | 0.010 |  |  |  |  |  |  |  |
|  |  | Peripheral stimulated intercept | 0.062 | 0.038 | 0.11 |  |  |  |  |  |  |  |
|  |  | **Central stimulated spinal cord** | -0.0055 | 0.0025 | 0.037 |  |  |  |  |  |  |  |
|  | **TLR4 agonist stimulation only** | Peripheral stimulated min | 0.015 | 0.010 | 0.15 | 5.42 | 34 | 4.9 | 31 | 0.0081 | 0.37 | 0.0029 |
|  |  | Peripheral stimulated intercept | -0.010 | 0.0058 | 0.089 . |  |  |  |  |  |  |  |
|  |  | Peripheral stimulated slope | -0.014 | 0.0079 | 0.094 |  |  |  |  |  |  |  |
|  | **Basal** | Spinal cord supernatant | 0.0034 | 0.0024 | 0.172 | 5.42 | 34 | 4.57 | 32 | 0.10 | 0.07 | 0.0096 |
|  |  | **Spinal cord** | 0.0050 | 0.0024 | 0.043 |  |  |  |  |  |  |  |
| **Neuronal and subcutaneous:** | **Complete** | Peripheral non-stimulated cell count . | -3.47e-08 | 1.7e-08 | 0.075 | 4.51777 | 23 | 0.68 | 8 | 0.56 | 0.06 |  |
|  |  | **Peripheral TLR4 stimulated max** | 1.15e-01 | 3.54e-02 | 0.012 |  |  |  |  |  |  |  |
|  |  | **Peripheral TLR4 stimulated min** | 1.82e-01 | 5.077e-02 | 0.0071 |  |  |  |  |  |  |  |
|  |  | **Peripheral TLR4 stimulated intercept** | -2.89e-01 | 8.49e-02 | 0.0093 |  |  |  |  |  |  |  |
|  |  | **Peripheral TLR4 stimulated slope** | -2.82e-01 | 8.053e-02 | 0.0080 |  |  |  |  |  |  |  |
|  |  | **Peripheral TLR2 stimulated max** | -1.53e-01 | 6.43e-02 | 0.044 |  |  |  |  |  |  |  |
|  |  | Peripheral TLR2 stimulated min | 8.98e-02 | 1.04e-01 | 0.41 |  |  |  |  |  |  |  |
|  |  | Peripheral TLR2 stimulated intercept | 1.11e-01 | 1.2e-01 | 0.38 |  |  |  |  |  |  |  |
|  |  | Peripheral TLR2 stimulated slope | 2.16e-01 | 1.39e-  01 | 0.16 |  |  |  |  |  |  |  |
|  |  | **Central TLR4 stimulated spinal cord supernatant** | 1.016e-01 | 4.12e-02 | 0.039 |  |  |  |  |  |  |  |
|  |  | **Central TLR2 stimulated spinal cord supernatant** | -1.64e-01 | 5.62e-02 | 0.02 |  |  |  |  |  |  |  |
|  |  | **Central non-stimulated spinal cord supernatant** | 3.5e-02 | 1.38e-02 | 0.035 |  |  |  |  |  |  |  |
|  |  | Central TLR4 stimulated spinal cord | -6.59e-03 | 4.93e-03 | 0.22 |  |  |  |  |  |  |  |
|  |  | **Central TLR2 stimulated spinal cord** | -1.023e-02 | 4.16e-03 | 0.039 |  |  |  |  |  |  |  |
|  |  | **Central non-stimulated spinal cord** | 1.53e-02 | 5.35e-03 | 0.021 |  |  |  |  |  |  |  |
|  |  |  |  |  |  |  |  |  |  |  |  |  |
|  |  |  |  |  |  |  |  |  |  |  |  |  |
|  | **Periphery** | **Non-stimulated cell count** | -3.44e-08 | 1.67e-08 | 0.05 | 5.52 | 29 | 3.38 | 24 | 0.29 | 0.04 | 0.15 |
|  |  | **Non-stimulated cells** | 7.89e-02 | 3.24e-02 | 0.022 |  |  |  |  |  |  |  |
|  |  | **TLR4 stimulated max** | -1.81e-02 | 6e-03 | 0.0059 |  |  |  |  |  |  |  |
|  |  | **TLR4 stimulated intercept** | 1.65e-02 | 6.12e-03 | 0.013 |  |  |  |  |  |  |  |
|  |  | TLR4 stimulated min | -7.053e-02 | 4.71e-02 | 0.15 |  |  |  |  |  |  |  |
|  | **Central** | TLR4 stimulated spinal cord supernatant | -0.016 | 0.012 | 0.19 | 4.52 | 23 | 2.46 | 19 | 0.34 | 0.02 | 0.19 |
|  |  | Non-stimulated spinal cord supernatant | 0.0049 | 0.0025 | 0.070 . |  |  |  |  |  |  |  |
|  |  | **TLR2 stimulated spinal cord** | -0.0077 | 0.0032 | 0.029 |  |  |  |  |  |  |  |
|  |  | **Non-stimulated spinal cord** | 0.011 | 0.0033 | 0.0039 |  |  |  |  |  |  |  |
|  | **TLR2 agonist stimulation only** | **Peripheral stimulated max** | -0.041 | 0.017 | 0.031 | 4.52 | 23 | 2.24 | 18 | 0.37 | 0.02 | 0.2 |
|  |  | Peripheral stimulated min | -0.073 | 0.058 | 0.23 |  |  |  |  |  |  |  |
|  |  | **Peripheral stimulated intercept** | 0.084 | 0.038 | 0.041 |  |  |  |  |  |  |  |
|  |  | **Central stimulated spinal cord** | 0.0091 | 0.0033 | 0.013 |  |  |  |  |  |  |  |
|  | **TLR4 agonist stimulation only** | Peripheral stimulated min | 0.018 | 0.0136 | 0.2 | 4.52 | 23 | 4.0018 | 20 | -0.019 | 0.48 | 0.052 . |
|  |  | Peripheral stimulated intercept | -0.014 | 0.0087 | 0.13 |  |  |  |  |  |  |  |
|  |  | Peripheral stimulated slope | -0.019 | 0.013 | 0.15 |  |  |  |  |  |  |  |
|  | **Basal** | Peripheral cells | 0.029 | 0.021 | 0.18 | 4.52 | 23 | 3.03 | 20 | 0.23 | 0.04 | 0.12 |
|  |  | Central spinal cord supernatant | 0.0046 | 0.0026 | 0.096 . |  |  |  |  |  |  |  |
|  |  | **Central spinal cord** | 0.010 | 0.0036 | 0.010 |  |  |  |  |  |  |  |
| **Neuronal Only:** | **Complete** | Peripheral non-stimulated plasma | -0.013 | 0.007 | 0.088. | 3.12 | 20 | 0.56 | 11 | 0.67 | 0.0048 |  |
|  |  | **Peripheral non-stimulated cells** | 0.12 | 0.027 | 0.00094 |  |  |  |  |  |  |  |
|  |  | **Peripheral TLR4 stimulated max** | -0.024 | 0.0089 | 0.022 |  |  |  |  |  |  |  |
|  |  | **Peripheral TLR4 stimulated slope** | 0.068 | 0.027 | 0.03 |  |  |  |  |  |  |  |
|  |  | **Peripheral TLR2 stimulated max** | -0.037 | 0.014 | 0.022 |  |  |  |  |  |  |  |
|  |  | Central TLR4 stimulated spinal cord supernatant | -0.023 | 0.015 | 0.15 |  |  |  |  |  |  |  |
|  |  | Central non-stimulated spinal cord supernatant | 0.013 | 0.0096 | 0.21 |  |  |  |  |  |  |  |
|  |  | Central TLR2 stimulated spinal cord | -0.0045 | 0.003 | 0.16 |  |  |  |  |  |  |  |
|  |  | Central non-stimulated spinal cord | 0.0042 | 0.0021 | 0.065 . |  |  |  |  |  |  |  |
|  | **Periphery** | Non-stimulated plasma | -0.017 | 0.0081 | 0.051 . | 3.4 | 22 | 1.068 | 15 | 0.62 | 0.0038 | 0.16 |
|  |  | **Non-stimulated cells** | 0.12 | 0.025 | 0.00028 |  |  |  |  |  |  |  |
|  |  | **TLR4 stimulated max** | -0.02 | 0.0074 | 0.019 |  |  |  |  |  |  |  |
|  |  | **TLR4 stimulated slope** | 0.064 | 0.023 | 0.012 |  |  |  |  |  |  |  |
|  |  | **TLR2 stimulated max** | -0.034 | 0.016 | 0.05 |  |  |  |  |  |  |  |
|  |  | TLR2 stimulated min | -0.087 | 0.049 | 0.096 . |  |  |  |  |  |  |  |
|  |  | TLR2 stimulated slope | -0.061 | 0.040 | 0.15 |  |  |  |  |  |  |  |
|  | **Central** | **TLR2 stimulated spinal cord** | -0.0078 | 0.0035 | 0.038 | 3.11 | 20 | 2.35 | 18 | 0.16 | 0.078 | 0.0089 |
|  |  | Non-stimulated spinal cord | 0.005 | 0.0029 | 0.1 |  |  |  |  |  |  |  |
|  | **TLR2 agonist stimulation only** | Central spinal cord - | 0.0056 | 0.0034 | 0.12 | 3.11 | 20 | 2.73 | 19 | 0.076 | 0.12 | 0.0063 |
|  | **TLR4 agonist stimulation only** | N/A |  |  |  |  |  |  |  |  |  |  |
|  | **Basal** | Peripheral cell count | -2.86e-08 | 2.26e-08 | 0.22 | 3.11 | 20 | 1.63 | 16 | 0.35 | 0.027 | 0.022 |
|  |  | Peripheral plasma | -1.29e-02 | 9.89e-03 | 0.22 |  |  |  |  |  |  |  |
|  |  | **Peripheral cell** | 7.66e-02 | 2.79e-02 | 0.014 |  |  |  |  |  |  |  |
|  |  | Central spinal cord supernatant | -1.267e-02 | 6.041e-03 | 0.052 . |  |  |  |  |  |  |  |

Notes: Significant variables are shown in bold. One-way ANOVA was used to compare which subsets (Central/ Peripheral/ TLR2/ TLR4 or Basal) when compare with all outputs is a better model. The residual deviance for the model includes predictor variables, whereas the null deviance for the model does not. SE, standard error.

**Table S3. Best-fit logistic regression model from rats (Peripheral only) and from humans to predict the presence of pain in chronic pain patients.**

|  | **Variables** | **Estimate** | **SE** | **p-value** | **Null deviance** | **df** | **Residual deviance** | **Df** | **AUC** | **ANOVA** |
| --- | --- | --- | --- | --- | --- | --- | --- | --- | --- | --- |
| **Model rat to human** | Non-stimulated cell count | -4.04e-1 | 2.67 | 0.88 | 35.59 | 26 | 16.68 | 21 | 0.94 |  |
|  | TLR4 stimulated max | 4.52e-8 | 2.11e-7 | 0.83 |  |  |  |  |  |  |
|  | TLR4 stimulated intercept | 5.55e-3 | 3.93 | 0.16 |  |  |  |  |  |  |
|  | TLR2 stimulated max | 5.32e-3 | 2.66e-3 | 0.045* |  |  |  |  |  |  |
|  | TLR2 stimulated min | -1.63e-1 | 1.26e-1 | 0.195 |  |  |  |  |  |  |
| **Model human** | TLR4 stimulated max | -0.0038 | 0.0031 | 0.22 | 35.59 | 26 | 18.16 | 23 | 0.92 | NS |
|  | TLR4 stimulated intercept | 0.0042 | 0.003 | 0.16 |  |  |  |  |  |  |
|  | TLR2 stimulated max | 0.0045 | 0.0023 | 0.054 |  |  |  |  |  |  |

Notes: The residual deviance for the model includes predictor variables, whereas the null deviance for the model does not. The discrimination probabilities (D, area under ROC curve) are presented in the table. One-way ANOVA was used to compare Model human with Model rat to human. The residual deviance for the model includes predictor variables, whereas the null deviance for the model does not. SE, standard error.
